# Mailed Outreach and Patient Navigation for Colorectal Cancer Screening Among Rural Medicaid Enrollees: A Cluster Randomized Clinical Trial

**DOI:** 10.1001/jamanetworkopen.2025.0928

**Published:** 2025-03-17

**Authors:** Gloria D. Coronado, Amanda F. Petrik, Michael C. Leo, Jennifer Coury, Robert Durr, Brittany Badicke, Jamie H. Thompson, Anna C. Edelmann, Melinda M. Davis

**Affiliations:** 1 University of Arizona Cancer Center, Tucson; 2 Kaiser Permanente Center for Health Research, Portland, Oregon; 3 Oregon Rural Practice-Based Research Network, Portland

## Abstract

**Importance:**

Approximately 60 million adults live in rural regions of the US, which historically have low rates of colorectal cancer (CRC) screening and follow-up. Rural residents enrolled in Medicaid have particularly low CRC screening and follow-up rates.

**Objective:**

To determine the effectiveness and implementation of a collaborative Medicaid health plan–clinic program of mailed fecal immunochemical test (FIT) outreach and patient navigation to colonoscopy following an abnormal FIT result when implemented in rural clinics as part of standard care.

**Design, Setting, and Participants:**

This cluster randomized clinical trial was conducted at 28 rural clinic units in Oregon affiliated with 3 Medicaid health plans. The clinics were randomized to the intervention (n = 14) or to usual care (n = 14). Participants were Medicaid enrollees (aged 50-75 years) due for CRC screening. The intervention was delivered from May 11, 2021, through June 4, 2022, and analyses were performed from June 2023 through September 2024.

**Intervention:**

The stepwise intervention involved (1) mailed FIT outreach and (2) patient navigation to colonoscopy following an abnormal FIT result. Implementation support included practice facilitation, training, collaborative learning, and patient tracking tools.

**Main Outcomes and Measures:**

The primary effectiveness outcome was completion of any CRC screening within 6 months of eligibility determination. An additional effectiveness outcome was follow-up colonoscopy completion within 6 months of an abnormal FIT result. Implementation was measured as (1) the proportion of intervention-eligible enrollees who were mailed an FIT and who were sent an advance notification or reminder and (2) the proportion with an abnormal FIT result who were offered patient navigation.

**Results:**

This study included 5614 Medicaid enrollees (2613 in intervention clinics and 3001 in usual care clinics). Enrollees had a mean (SD) age of 58.2 (5.5) years; most (4940 [88.0%]) were aged 50 to 64 years. A total of 2948 enrollees (52.5%) were female, 325 (6.2%) were Hispanic and 3774 (67.2%) were White, and 4457 (79.4%) lived in rural regions. Compared with Medicaid enrollees in usual care clinics, enrollees in intervention clinics had a higher adjusted 6-month proportion of any CRC screening completion (11.8% vs 4.5%; difference, 7.3 [95% CI, 5.3-9.2] percentage points). Implementation was 100% (all 1489 intervention-eligible enrollees) for mailed FIT outreach, 88.5% for advance notification, 78.1% for reminders, and 57.9% for patient navigation.

**Conclusions and Relevance:**

In this cluster randomized clinical trial of rural clinics, mailed FIT outreach and patient navigation boosted participation in CRC screening among Medicaid enrollees. More efforts are needed to address low participation in both FIT testing and follow-up colonoscopy.

**Trial Registration:**

ClinicalTrials.gov Identifier: NCT04890054

## Key Points

**Question:**

How effective is a program of mailed fecal immunochemical test (FIT) outreach and patient navigation to colonoscopy following an abnormal FIT result when implemented in primary care clinics serving rural Medicaid enrollees?

**Findings:**

In this cluster randomized clinical trial of 5614 Medicaid enrollees at 28 rural clinics in Oregon, a mailed FIT outreach and patient navigation program led to a significant 7.3–percentage point increase in colorectal cancer (CRC) screening over usual care.

**Meaning:**

Mailed FIT outreach and patient navigation boosted CRC screening in the rural primary care clinics studied.

## Teaser

This cluster randomized clinical trial evaluates colorectal cancer screening among Medicaid enrollees in rural clinics in Oregon via mailed fecal immunochemical test (FIT) outreach and patient navigation to colonoscopy after an abnormal result.

## Introduction

Screening for colorectal cancer (CRC) reduces CRC incidence and mortality.^1,2,3^ Since 2008, the US Preventive Services Task Force has recommended CRC screening for adults aged 50 to 75 years; in 2021, the guidelines were updated to recommend starting at age 45 years.^4,5^ However, screening participation is suboptimal in the US, particularly among individuals residing in rural regions or who are enrolled in Medicaid.^6,7,8^ Research is needed to identify ways to realize the full potential of routine screening to detect and prevent CRC among these populations.

Systematic reviews and meta-analyses have shown that mailing fecal immunochemical test (FIT) kits to individuals who are due for CRC screening can improve screening rates by 22 to 28 percentage points.^9,10,11^ However, few studies have evaluated the effectiveness or implementation of mailed FIT programs in rural geographic regions of the US or among Medicaid-enrolled adults.^12^

Patient navigation has also been shown to improve rates of CRC screening and follow-up after abnormal test results. In a 2020 meta-analysis, researchers reported average relative improvements in CRC screening of 64% over usual care following implementation of patient navigation.^13^ Importantly, however, of the 22 randomized trials included in that review, only 2 were conducted in rural regions.^14,15^ Two reviews have shown that among evidence-based interventions, the combination of mailed FIT outreach and patient navigation leads to the greatest improvement in CRC screening.^9,16^ Despite this evidence, the adoption of combined mailed FIT and patient navigation programs has been slow; it has been particularly slow in rural clinics, where patients may have low awareness of stool-based test options and less engagement with health care services than individuals in urban regions.^17,18^

The SMARTER CRC (Screening More Patients for CRC Through Adapting and Refining Targeted Evidence-Based Interventions in Rural Settings) trial evaluated the implementation and effectiveness of a mailed FIT outreach and patient navigation program delivered as a partnership among the research team, Medicaid health plans, and rural clinics.^19^ This article presents primary and secondary outcomes from the study, including evaluations of the intervention’s effectiveness at boosting CRC screening and follow-up as well as implementation success across participating clinics.

## Methods

The SMARTER CRC cluster randomized clinical trial was conducted as part of the National Cancer Institute (NCI)–funded Accelerating Colorectal Cancer Screening and Follow-Up Through Implementation Science (ACCSIS) consortium (NCI Clinical Trials Reporting Program: NCI-2021-01032).^20^ The trial is a partnership between the Oregon Health & Science University (OHSU) Oregon Rural Practice-Based Research Network and the Kaiser Permanente Center for Health Research. The OHSU Institutional Review Board approved the trial and granted a waiver of informed consent because the study involved minimal risk to the participants. This study follows the Consolidated Standards of Reporting Trials (CONSORT) reporting guideline.

The SMARTER CRC study design, setting, protocol, and outcomes have been described previously.^19^ The study protocol is available in Supplement 1. Briefly, SMARTER CRC was a large-scale, parallel, 2-group cluster randomized clinical trial that involved 3 Medicaid health plans and 29 rural clinic units (ie, individual clinics or groups of clinics), followed by a scale-up study involving 130 rural clinics.^21^ Clinic units were randomized to implement the intervention or to continue providing usual care. The intervention was tailored for rural Medicaid populations,^22^ with components delivered by health plans, clinics, and direct-mail vendors. The intervention was delivered from May 11, 2021, through June 4, 2022.

### Setting

We recruited 3 health plans that serve rural counties in Oregon (33.3% of the 9 invited).^19,23^ The research team and participating health plans recruited affiliated clinics that had at least 30 age-eligible Medicaid enrollees or individuals dually enrolled in Medicaid and Medicare (dual enrollees are aged <65 years and have a disability, end-stage kidney disease, or amyotrophic lateral sclerosis), had CRC screening rates of 60% or lower, and operated in a geographic region designated as rural or frontier.^24,25^ A total of 33 clinics (56.9% of the 58 clinics invited), organized into 29 clinic units for randomization, were recruited between January 2020 and April 2021.^19^

### Clinic Randomization

The project statistician stratified allocation assignments by clinic unit affiliation (hospital affiliated, health care network affiliated, clinic, multiple locations, or single location) and randomized clinic units to either usual care (n = 14) or the intervention (n = 15) in 2 batches in April 2021 (2 health plans) and May 2021 (1 health plan), with timing based on data availability. Shortly after randomization, 1 intervention clinic closed (patients were absorbed in other study clinics), leaving 14 intervention clinic units. Randomization was performed using Stata, version 17 (StataCorp LLC). Neither researchers nor clinic staff were blinded to randomization assignment.

### Enrollee Eligibility

Health plan staff used claims data to identify enrollees who were aged 50 to 75 years, enrolled in Medicaid or dually enrolled in Medicaid and Medicare, and had no disqualifying health condition based on codes used in prior studies and described in the trial protocol.^26,27^ This led to the identification of 5614 individuals (2613 in intervention clinics and 3001 in usual care clinics (Figure) included in primary end-point analysis. Claims lists were pulled from May to July 2021, then entered into a REDCap (research electronic data capture) database.^28,29^ From May to September 2021, clinic staff in intervention clinics then used electronic health record (EHR) data to review the resulting lists, removing any enrollee who was not due for CRC screening, was ineligible for FIT testing, or had not established or was no longer receiving care at the clinic.

**Figure.**
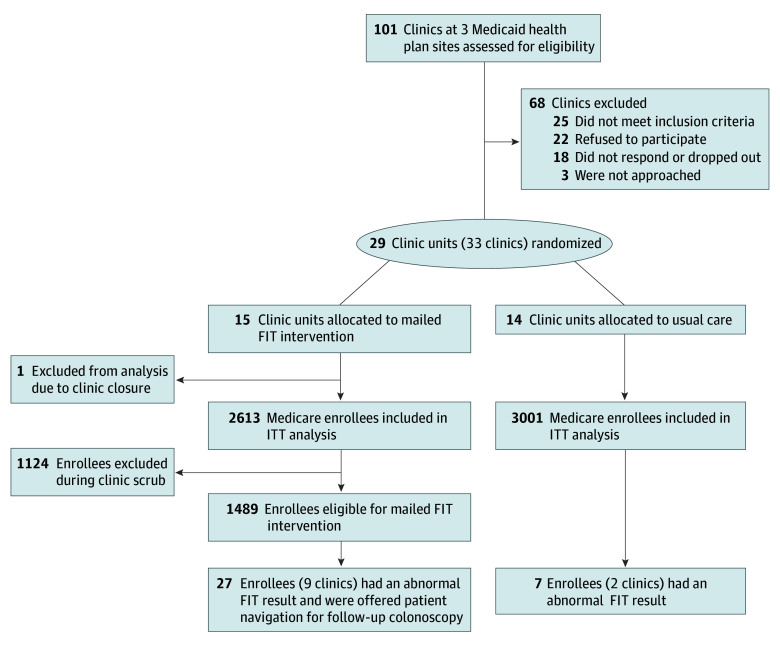
Patient Flowchart Among the 5614 eligible enrollees, 246 (4.4%) disenrolled within 6 months and 421 (7.5%) disenrolled within 12 months and had no evidence of colorectal cancer screening completion; all were included in the primary analysis. FIT indicates fecal immunochemical testing; ITT, intention to treat.

### Usual Care

Participating health plans had a variety of methods for promoting CRC screening and all had implemented mailed FIT outreach programs in some clinics, as previously described.^19^ Clinics generally offered opportunistic CRC screening to patients attending clinic visits. Clinic staff contacted patients with abnormal FIT results and referred them to clinicians offering colonoscopy services in the clinic’s region. The intervention was overlaid on these existing screening promotion efforts.

### Intervention Activities

#### Mailed FIT Outreach

Vendors (PrintSync Inc or Koko Graphix) for 2 health plans (9 clinics) mailed introduction letters to all intervention-eligible enrollees, followed 1 week later by an FIT. A vendor (Home Access Health Corporation) for the third health plan did not mail a separate introduction letter. Mailed with the FIT kit were a wordless FIT instruction sheet^30^ (1 health plan), a letter with information about FIT testing and instructions on how to return the completed test (all health plans), and a preaddressed and postage-paid envelope (all health plans). The mailed FIT kit was the same as that used in the clinic; all kits used standard positivity thresholds set by the manufacturers. The kits included the following: the OC-Auto FIT (PolyMedco), the OC-Light S FIT (PolyMedco), the InSure ONE FIT (Clinical Genomics), the Hemosure iFOB Test Kit (Hemosure Inc), and the Henry Schein OneStep+ test (Henry Schein Inc). Health plans 2 and 3 also delivered a reminder to complete the FIT (via text message or letter) approximately 2 weeks after the FIT mailing. Intervention clinic staff were encouraged to conduct outreach to enrollees either before or after the FIT mailing.^31^

#### Patient Navigation

The patient navigation intervention for follow-up colonoscopy was adapted from the New Hampshire Colorectal Cancer Screening Program^32^ based on prior implementations by our study team and pilot testing for rural clinics.^33,34^ Our patient navigation intervention was telephone based, could be delivered by clinic or health plan staff, and covered 4 topics: barriers to assessment or resolution, colonoscopy preparation, colonoscopy reminder, and results reporting. Patient navigators were trained by the research team.^35^ Navigation activities were tracked in the study database.

#### Implementation Support

Implementation facilitation and training are described elsewhere.^19^ Briefly, practice facilitators on the research team held two 1-hour introductory meetings and maintained ongoing contact with clinical staff to address challenges and provide support. A collaborative learning meeting was held monthly with members of the research team, health plan representatives, and staff from all intervention clinics; it served to facilitate collaboration and booster training and identify needed adaptations to the intervention.

The patient navigation training was offered during the summer of 2021 to at least 1 staff member in each intervention clinic unit and to 1 health plan staff member (a community health worker from health plan 1). Training covered how to review patient lists for mailed FIT eligibility (2 identical 60-minute virtual sessions with 1 to 2 staff members from each clinic, provided live or as a recording) and how to conduct patient navigation (one 5-hour training made up of prerecorded videos and live interactive webinars).^34^

### Evaluation

To obtain data for outcomes that were unavailable in claims data (FIT result, etc), research team members (eg, practice facilitators, data analysts) conducted EHR audits of enrollees who were identified as having completed CRC screening (based on claims data, vendor data, and REDCap data). These research team members gathered FIT results, as well as colonoscopy outcomes for those with abnormal FIT results (colonoscopy date, presence of adenomas or cancer). For practical reasons, these research team members were unblinded to study allocation and worked directly with clinic staff to gather data from EHRs.

#### Effectiveness

The primary effectiveness outcome was whether enrollees in the intervention clinic units were more likely than those in usual care clinic units to obtain any CRC screening within 6 months (and secondarily at 12 months) of the claims list pull date. Additional effectiveness outcomes included FIT completion at 6 and 12 months; among those with an abnormal FIT result, we assessed follow-up colonoscopy completion within 6 and 12 months and time to colonoscopy. We also report colonoscopy outcomes (number of adenomas or cancers detected). Primary analyses relied on intention-to-treat principles: enrollees remained in randomized intervention or usual care groups regardless of whether they received the intervention (including enrollees who were removed during the review process or transferred to another clinic during follow-up). For all analysis, eligible individuals who disenrolled in Medicaid during the evaluation intervals and had no claim for CRC screening (246 enrollees in 6-month data and 421 in 12-month data) were assumed to have not completed CRC screening. We performed sensitivity analyses assessing intervention effectiveness in models that coded these disenrolled individuals’ screening outcomes as missing.

#### Implementation

Our implementation outcomes were the proportion of intervention-eligible enrollees who were mailed an FIT, were sent an advance notification or reminder, and were offered patient navigation within 6 months of eligibility determination.

### Statistical Analysis

Our preplanned analysis used hierarchical generalized linear modeling to account for the intraclass correlation resulting from the clustering of enrollees within clinic units.^36^ We chose this approach over generalized estimating equations (GEEs) because the former can produce both population-average and cluster-specific effects, whereas GEEs only provide population-average effects. The independent variable was study condition, with usual care as the reference group. Clinic unit cluster was modeled as a random effect. We compared adjusted mean time of obtaining a colonoscopy among enrollees who completed a follow-up colonoscopy in each group. All models included Medicaid health plan, individual age, and sex as covariates because of their known relationship to the outcomes.^37,38^ Race and ethnicity were determined based on administrative claims records; these data are included because of their known association with mailed FIT outreach. Ethnicity is reported as Hispanic, and race is reported as White, other or multiple races (including American Indian or Alaska Native, Asian, Black, and Native Hawaiian or Other Pacific Islander, which were grouped together owing to small numbers in each category), or unknown or not reported.

All analyses were 2-tailed and considered significant if *P* < .05. Power calculations were derived using PASS, version 15 (NCSS Statistical Software),^39^ and showed that a 10–percentage point change in screening rates could be detected, assuming 106 patients per clinic, an intraclass correlation coefficient of 0.03, and a baseline screening rate of 44.6%, based on prior research and baseline rates.^19,40^ Reported proportions are predicted estimates (rather than crude No. [%] values) generated using marginal standardization and accounting for clustering and covariates. Observed values are reported as the No. (%) of patients with an abnormal FIT result. Analyses were performed from June 2023 through September 2024.

## Results

### Clinic Characteristics

Characteristics of the 28 intervention and usual care clinic units are shown in Table 1. The 14 intervention clinic units were predominantly small, hospital-affiliated rural health clinics. The 14 usual care clinic units were slightly larger on average and had a mix of federal designations and affiliations.

**Table 1.** Characteristics of Participating Clinics and Patients in the Analytic Sample

Characteristic	Patient group	
	Intervention (n = 14)^a^	Usual care (n = 14)
**Clinic unit level (n = 28), No. (%)^b^**		
Federal designation		
Rural health clinic	7 (50.0)	5 (35.7)
Federally qualified health center	1 (7.1)	4 (28.6)
Tribal health center	1 (7.1)	0
No federal designation	5 (35.8)	5 (35.7)
Clinic affiliation or network structure		
Hospital affiliated	8 (57.2)	5 (35.7)
Health care network affiliated	2 (14.3)	2 (14.3)
Clinic with multiple locations	1 (7.1)	5 (35.7)
Individual clinic (single location)	3 (21.4)	2 (14.3)
Health plan affiliation		
Health plan 1	8 (57.2)	6 (42.9)
Health plan 2	5 (35.7)	7 (50.0)
Health plan 3	1 (7.1)	1 (7.1)
No. of eligible patients per clinic		
<100	8 (57.2)	4 (28.6)
100-199	3 (21.4)	7 (50.0)
≥200	3 (21.4)	3 (21.4)
**Patient level (N = 5614), clinic median (range), %**		
Age, y		
50-54	27.6 (14.5-40.6)	28.9 (19.0-40.0)
55-59	27.6 (24.2-44.7)	27.8 (20.0-34.1)
60-64	25.9 (20.4-42.2)	28.6 (20.1-38.3)
65-74	17.5 (2.2-23.1)	15.6 (2.2-23.1)
Sex		
Female	54.7 (45.7-63.2)	57.1 (44.3-70.0)
Male	45.4 (36.8-54.3)	44.4 (30.0-55.7)
Race		
White	77.1 (4.3-87.5)	64.7 (48.4-84.1)
Other race or multiple races^c^	5.5 (2.3-85.1)	4.6 (0-7.5)
Unknown or not reported	18.4 (6.3-48.9)	32.6 (11.5-50.5)
Ethnicity		
Hispanic	2.9 (0-24.4)	4.4 (0-31.9)
Preferred language		
English	96.8 (80.0-100)	95.6 (67.0-100)
Spanish	1.8 (0-20.0)	2.1 (0-33.0)
Other or unknown	0.7 (0-14.3)	1.5 (0-5.0)
Rurality, based on Oregon Office of Rural Health designations		
Urban	0.2 (0-26.5)	2.1 (0-28.0)
Rural	97.2 (0-100)	79.8 (0.7-99.6)
Frontier	0 (0-99.3)	0 (0-98.5)
Missing or not in Oregon	0 (0-4.4)	0.1 (0-3.6)
Insurance status		
Medicaid	74.3 (54.2-100)	74.6 (67.2-100)
Dual Medicaid and Medicare	25.8 (0-45.8)	25.5 (0-32.8)
No. of clinic visits in 2021		
0	40.8 (17.9-62.9)	38.1 (20.1-55.6)
1	10.5 (6.1-17.2)	12.0 (7.7-17.5)
2-5	29.9 (15.7-51.9)	29.5 (20.0-42.7)
≥6	18.9 (8.8-31.1)	19.3 (9.8-34.1)
Ever had prior colorectal cancer screening		
Yes	19.0 (10.8-35.2)	14.1 (2.0-30.9)

^a^ One of the original 15 clinics closed, resulting in 14 clinics; patients were absorbed by other clinics.

^b^ Percentages do not sum to 100 because values are median clinic percentages; percentages are weighted by clinic size.

^c^ Includes American Indian or Alaska Native, Asian, Black, Native Hawaiian or Other Pacific Islander, other race and ethnicity, or multiple races or ethnicities. Race and ethnicity was based on administrative claims records.

### Enrollee Characteristics

The primary evaluation included 5614 Medicaid enrollees (2613 in intervention clinics and 3001 in usual care clinics) (Figure). Enrollees had a mean (SD) age of 58.2 (5.5) years, and most (4940 [88.0%]) were aged 50 to 64 years. A total of 2948 enrollees (52.5%) were female and 2666 (47.5%) were male; 4457 (79.4%) lived in rural regions. With regard to race and ethnicity, 325 enrollees (6.2%) were Hispanic, 3774 (67.2%) were White, and 313 (5.6%) were of other or multiple races; race was unknown or not reported for 1527 enrollees (27.2%). Enrollee characteristics were similar across intervention and usual care conditions (Table 1). The median proportion of enrollees who had dual Medicaid and Medicare coverage was 25.4% overall (range, 0-45.8%). The median proportion of enrollees who had completed prior CRC screening was 19.0% (range, 10.8%-35.2%) in intervention clinic units and 14.2% (range, 2.0%-30.9%) in usual care clinic units. Individual-level characteristics, characteristics of individuals who disenrolled, and rural designations are provided in eTables 1, 2, and 3 in Supplement 2, respectively.

### Primary Outcome

The adjusted proportion of 6-month any CRC screening was 7.3 (95% CI, 5.3-9.2) percentage points higher (*P* < .001) for enrollees in clinic units allocated to intervention vs usual care (11.8% vs 4.5%) (Table 2). The residual intraclass correlation coefficient was 0.008 (95% CI, <0.001-0.07).

**Table 2.** CRC Screening Completion Among the Intention-to-Treat Sample, by Intervention and Usual Care Groups

Outcome	Patient group		Percentage point difference, mean (95% CI)	*P* value^a^
	Intervention	Usual care		
**CRC screening **				
Within 6 mo of list pull^b^				
No. of enrollees initially eligible	2613	3001	NA	NA
Completed any CRC screening, %^c^	11.8	4.5	7.3 (5.3-9.2)	<.001
Completed FIT, %^c^	8.5	2.2	6.3 (4.6-8.1)	<.001
Within 12 mo of list pull^d^				
No. of enrollees eligible	2613	3001	NA	NA
Completed any CRC screening, %^c^	16.8	9.0	7.8 (6.0-9.6)	<.001
Completed FIT, %^c^	11.5	4.5	7.0 (4.6-9.4)	<.001
**Follow-up colonoscopy**				
Within 6 mo of abnormal FIT result				
No. of enrollees with an abnormal FIT result	27	7	NA	NA
Completed colonoscopy, %^c^	43.3	15.4	27.9 (1.2-54.6)	.04
Time from abnormal FIT, mean (95% CI), d^e^	96.3 (72.6- 119.9)	114.6 (50.0-179.2)	−18.4 (−89.4 to 52.7)	.61
Within 12 mo of abnormal FIT result				
No. of enrollees with an abnormal FIT result	27	7	NA	NA
Completed colonoscopy, %^c^	49.1	19.7	29.4 (−2.2 to 60.9)	.07
Time from abnormal FIT, mean (95% CI), d^e^	115.8 (95.8- 135.8)	110.5 (54.1-166.9)	5.3 (−56.4 to 67.1)	.87

Abbreviations: CRC, colorectal cancer; FIT, fecal immunochemical test; NA, not applicable.

^a^ Two-sided significance level based on multilevel logistic regression adjusted for sex, age, Medicaid health plan, and intraclass correlation within clinic.

^b^ Screening outcome was coded as “no” for individuals who disenrolled in Medicaid and had no claims evidence of CRC screening during the 6-month evaluation interval (246 of 5614 [4.4%]: 132 of 2613 [5.1%] in intervention clinics and 114 of 3001 [3.8%] in usual care clinics).

^c^ Proportions are predicted estimates (rather than crude No. [%] values) generated using marginal standardization and accounting for clustering and covariates (sex, age, and Medicaid health plan).

^d^ Screening outcome was coded as “no” for individuals who disenrolled in Medicaid and had no claims evidence of CRC screening during the 12-month evaluation interval (421 of 5614 [7.5%]; 202 of 2613 [7.7%] in intervention clinics and 219 of 3001 [7.3%] in usual care clinics).

^e^ Adjusted means and differences based on multilevel regression adjusted for sex, age, Medicaid health plan, and intraclass correlation within clinic.

### Secondary Outcomes

Proportions of 6-month FIT completion were higher among enrollees in intervention vs usual care clinics (8.5% vs 2.2%; difference, 6.3 [95% CI, 4.6-8.1] percentage points; *P* < .001). In analyses of proportions of 12-month screening, between-group differences were 7.8 (95% CI, 6.0-9.6) percentage points and 7.0 (95% CI, 4.6-9.4) percentage points (both *P* < .001) for any CRC screening (16.8% vs 9.0%) and FIT testing (11.5% vs 4.5%), respectively. Among the enrollees who were mailed an FIT, the return rate was 12.2% (181 of 1489); among those who were mailed an FIT and sent a reminder, the return rate was 16.7% (149 of 893) at 6 months. Among the 281 enrollees (213 in intervention clinics and 68 in usual care clinics) who completed an FIT within 6 months, 34 (12.1%; 27 in intervention clinics and 7 in usual care clinics) had an abnormal result and were thus due for follow-up colonoscopy. Among the 34 enrollees, follow-up colonoscopy completion proportions differed by intervention condition in 6-month data (intervention, 43.3% vs usual care, 15.4%; difference, 27.9 [95% CI, 1.2-54.6] percentage points; *P* = .04) but did not significantly differ for 12-month data (intervention, 49.1% vs usual care, 19.7%; difference, 29.4 [95% CI, −2.2 to 60.9] percentage points; *P* = .07).

Compared with the usual care group, the adjusted mean time to follow-up colonoscopy completion among the intervention group was 18.4 (95% CI, −89.4 to 52.7) days shorter within 6 months (*P* = .61) and 5.3 (95% CI, −56.4 to 67.1) days longer within 12 months (*P* = .87) of an abnormal FIT result. Among the 34 enrollees with an abnormal FIT result, 14 completed a colonoscopy within 12 months; of these enrollees, 7 (50.0%) had an adenoma and 1 (7.1%) had cancer.

### Sensitivity Analyses

A total of 246 enrollees (4.4%; 132 [5.1%] in intervention clinics and 114 [3.8%] in usual care clinics) disenrolled in Medicaid within 6 months, had no documented evidence of CRC screening, and were therefore coded as having missing outcome data. The sensitivity analyses, conducted with the 5368 individuals with complete outcome data, yielded results similar to our primary analysis: the proportion of adjusted 6-month any CRC screening was 7.7 (95% CI, 5.8-9.6) percentage points higher (*P* < .001) for enrollees in clinic units allocated to intervention vs usual care (12.4% vs 4.7%). Similarly, 421 of 5614 enrollees (7.5%; 202 [7.7%] in intervention clinics and 219 [7.3%] in usual care clinics) disenrolled in Medicaid within 12 months and were coded as having missing outcome data. The sensitivity analyses, conducted with the 5207 individuals who had complete outcome data, yielded results similar to our primary analysis: the proportion of adjusted 12-month any CRC screening was 8.4 (95% CI, 6.5-10.4) percentage points higher (*P* < .001) for enrollees in clinic units allocated to intervention vs usual care (18.1% vs 9.7%).

### Implementation

Of the 2613 intervention enrollees identified as initially eligible using claims data, 1124 (43.0%) were determined to be ineligible during the review process (Table 3). All 1489 intervention-eligible enrollees were mailed an FIT. Implementation for other health plan–delivered components was high for introductory letters (3 health plans, 11 clinics, and 1318 [88.5%] eligible enrollees) and low for reminder letters (1 health plan, 5 clinics, and 310 [20.8%] eligible enrollees), and reminder text messages (1 health plan, 1 clinic, and 226 [38.9%] eligible enrollees). Implementation of clinic-delivered components was low for advance notification live calls (4 clinics, 29 enrollees) or text messages (1 clinic, 61 enrollees) and reminders delivered via live calls (8 clinics, 316 enrollees) or text messages (1 clinic, 54 enrollees) (Table 4). A total of 1318 eligible enrollees (88.5%) were sent at least 1 advance notification and 1163 (78.1%) were sent at least 1 reminder. Among the 1489 enrollees who were mailed an FIT, 181 (12.2%) completed it; 19 (10.5%) had an abnormal FIT result and were thus eligible for patient navigation. Of the 19 enrollees with an abnormal FIT result, 11 (57.9%) received patient navigation for at least 1 topic area.

**Table 3.** Implementation of Intervention Components Among Eligible Adults in Intervention Clinics at 6 Months

Intervention activity	No./total No. (%) of intervention clinic enrollees	No. of intervention clinic units (No. of enrollees per clinic, range)	Median clinic unit, % (range)
**Eligibility**			
Initially eligible (health plan claims)	2613	14 (32-1154)	100
Excluded during scrub (clinic)^a^	1124/2613 (43.0)	14 (10-575)	35.8 (15.6-71.4)
Expected for intervention components	1489/2613 (57.0)	14 (14-579)	57.0 (28.9-84.4)
**Step 1: Advance notification**			
Mailed introductory letter (health plan)	1318/1489 (88.5)	11 (29-579)	66.0 (28.9-84.4)
Delivered calls or texts (clinic)	90/1489 (6.0)	5 (1-61)	7.3 (0.5-100)
**Step 2: Mailed FIT outreach**			
Mailed FIT kit (health plan)	1489/1489 (100)	14 (14-579)	100
**Step 3: Reminders**			
Mailed reminder letter (health plan)	310/1489 (20.8)	5 (14-115)	100
Delivered live reminder call (clinic)	316/1489 (21.2)	8 (10-118)	56.5 (34.5-68.7)
Sent reminder text (health plan or clinic)	280/1489 (18.8)	2 (54-226)	47.7 (38.9-56.3)
Completed the FIT within 6 mo of identification	181/1489 (12.2)	14 (2-50)	12.6 (5.2-22.7)
Had an abnormal FIT result	19/181 (10.5)	7 (1-5)	12.5 (7.9-40.0)
**Step 4: Patient navigation for follow-up colonoscopy**			
Delivered patient navigation (clinic)	11/19 (57.9)	5 (1-5)	50.0 (33.3-100)

Abbreviation: FIT, fecal immunochemical test.

^a^ Clinic staff reviewed enrollee lists generated by health plans and excluded enrollees who were ineligible for colorectal cancer screening or had not established care.

**Table 4.** Implementation of Intervention Components Among Eligible Adults in Intervention Clinics at 6 Months, by Medicaid Health Plan and Clinic

Plan and clinic	Enrollees		Mailed FIT outreach components delivered^c^					FIT outcomes, No./total No. (%)^d^		Patient navigation among enrollees with an abnormal FIT result, No./total No. (%)	
	Initially eligible (claims), No.^a^	Eligible after clinic scrub, No./total No. (%)^b^	Advance notification, No./total No. (%)	Mailed FIT, No.	Reminder, No./total No. (%)					Patient navigation delivered	Follow-up colonoscopy completed
					Letter	Live calls (≥1)	Texts (≥1)	Completed FIT	Abnormal FIT result		
Enrollees (overall)	2613	1489/2613 (57.0)	1318/1489 (88.5)	1489^e^	310/1489 (20.8)^e^	316/1489 (21.2)	280/1489 (18.8)	181/1489 (12.2)	19/181 (10.5)	11/19 (57.9)	3/11 (27.3)
Health plan 1											
Clinic 1	223	90/223 (40.4)	90/90 (100)^f^	90^f^	0/90	0/90	0/90	15/90 (16.7)	3/15 (20.0)	0/3	0
Clinic 2	49	30/49 (61.2)	30/30 (100)	30	0/30	19/30 (63.3)	0/30	2/30 (6.7)	0/2	0	0
Clinic 3	39	29/39 (74.4)	29/29 (100)	29	0/29	10/29 (34.5)	0/29	4/29 (13.8)	0/4	0	0
Clinic 4	106	73/106 (68.9)	73/73 (100)	73	0/73	38/73 (52.1)	0/73	7/73 (9.6)	0/7	0	0
Clinic 5	145	96/145 (66.2)	96/96 (100)	96	0/96	0/96	54/96 (56.3)	5/96 (5.2)	0/5	0	0
Clinic 6	45	35/45 (77.8)	35/35 (100)	35	0/35	16/35 (45.7)	0/35	4/35 (11.4)	0/4	0	0
Clinic 7	256	216/256 (84.4)	216/216 (100)	216	0/216	118/216 (54.6)	0/216	38/216 (17.6)	3/38 (7.9)	1/3 (33.3)	0/1
Clinic 8	47	31/47 (66.0)	31/31 (100)	31	0/31	21/31 (67.7)	0/31	2/31 (6.5)	0/2	0	0
Total	910	600/910 (65.9)	600/600 (100)	600	0/600	222/600 (37.0)	54/600 (9.0)	77/600 (12.8)	6/77 (7.8)	1/6 (16.7)	0/1
Health plan 2											
Clinic 9	32	14/32 (43.8)	0/14	14^g^	14/14 (100)^g^	0/14	0/14	3/14 (21.4)	0/3	0	0
Clinic 10	159	113/159 (71.1)	0/113	113	113/113 (100)	0/113	0/113	12/113 (10.6)	1/12 (8.3)	0/1	0
Clinic 11	184	115/184 (62.5)	115/115 (100)	115	115/115 (100)	79/115 (68.7)	0/115	24/115 (20.9)	3/24 (12.5)	3/3 (100)	1/3 (33.3)
Clinic 12	83	24/83 (28.9)	24/24 (100)	24	24/24 (100)	14/24 (58.3)	0/24	5/24 (20.8)	2/5 (40.0)	1/2 (50.0)	0/1
Clinic 13	91	44/91 (48.4)	0/44	44	44/44 (100)	0/44	0/44	10/44 (22.7)	2/10 (20.0)	1/2 (50.0)	1/1 (100)
Total	549	310/549 (56.5)	139/310 (44.8)	310	310/310 (100)	94/310 (30.3)	0/310	54/310 (17.4)	8/54 (14.8)	5/8 (62.5)	2/5 (40.0)
Health plan 3											
Clinic 14	1154	579/1154 (50.2)	579/579 (100)^h^	579^h^	0/579	0/579	226/579 (38.9)^h^	50/579 (8.6)	5/50 (10.0)	5/5 (100)	1/5 (20.0)

Abbreviations: CRC, colorectal cancer; FIT, fecal immunochemical test.

^a^ Eligibility was determined based on Medicaid claims data.

^b^ Clinic staff reviewed claims lists and removed enrollees who were not eligible for CRC screening or who had not established care at the clinic.

^c^ Letters were sent before mailed FIT outreach to all those eligible following the clinic staff scrub. In addition to letters, live telephone calls were delivered to 7 (clinic 5), 1 (clinic 7), 1 (clinic 11), and 20 (clinic 12) enrollees; text messages were sent to 61 (clinic 5) enrollees.

^d^ Of 213 enrollees with completed FITs and 27 with abnormal FIT results in intervention clinics (intention-to-treat sample; Table 2), 32 and 8 enrollees were excluded during the clinic scrub, respectively. This left 181 enrollees who completed the FIT and 19 enrollees with an abnormal FIT result in the study database used by clinics.

^e^ Component delivered by health plans to all enrollees.

^f^ Component delivered by health plan 1.

^g^ Component delivered by health plan 2.

^h^ Component delivered by health plan 3.

## Discussion

In this trial, a multicomponent program of mailed FIT outreach and patient navigation to colonoscopy following an abnormal FIT result delivered as a collaborative partnership among research staff, health plans, and rural clinics, boosted participation in CRC screening at 6 months among Medicaid enrollees by 7.3 (95% CI, 5.3-9.2) percentage points compared with usual care. Our findings add to a growing body of literature on the effectiveness of mailed FIT outreach and underscore the continued need to address low participation in follow-up colonoscopy among individuals with abnormal stool-test results.

Although these results show promise, they represent smaller intervention effects than shown in previous efficacy studies. In a meta-analysis, Dougherty et al^9^ reported a 22–percentage point improvement in CRC screening in clinic-based programs, with higher improvements (26 percentage points) among studies with 6-month evaluation intervals (as in our study). A 2024 evaluation of the Moonshot Consortium SCORE program involved 2 health centers and reported a 20–percentage point improvement in CRC screening following a mailed FIT and patient navigation program delivered by centralized research staff vs usual care.^41^ The smaller effect in our trial may reflect characteristics of the setting or population,^12^ the efficacy-effectiveness gap, or both, in which the effect of an intervention in an idealized research setting is attenuated by real-life conditions when implemented within a health care system.^42^

Contributing to the attenuation is that a large share (4602 [82.0%]) of enrollees in this study had no prior history of CRC screening, which is known to predict relatively low response to mailed FIT outreach (this compares to 76% in the SCORE study).^43,44,45^ In addition, 43.0% of enrollees (1124 of 2613) in this study who were identified as eligible using claims data were determined to be ineligible following the clinic review process and were not mailed an FIT (most had not established care with the clinic). This exclusion proportion was higher than in a previous evaluation by our study team (18% were removed in our prior study, BeneFIT^27^) yet lower than in our pilot evaluation (55% were removed in the pilot^46^). These ineligible enrollees were included in our primary intention-to-treat analysis. In analysis limited to the 1489 enrollees who were mailed an FIT, the 6-month return rate was 12.2%; among those who were mailed an FIT and sent a reminder, the 6-month return rate was 16.7%. Future efforts should focus on implementation strategies to spur cancer screening uptake among Medicaid enrollees without an established primary care practice relationship.

Although we evaluated only 34 enrollees with abnormal FIT results (intention-to-treat data), our findings show that only 43.3% of these enrollees in intervention clinics completed a follow-up colonoscopy within 6 months; this compared to 15.4% in usual care clinics, a significant 27.9–percentage point (95% CI, 1.2-54.6) difference. Although the between-group difference was similar to that reported in the SCORE study (68.8% in intervention clinics and 44.4% in usual care clinics; difference, 24.4 percentage points^41^), our absolute proportions were much lower. Further efforts are needed to boost participation in follow-up colonoscopy, given that this study and prior research demonstrate suboptimal participation across settings,^47,48^ and individuals with abnormal stool-test results have a 5% or higher probability of having cancer.^49^

Workforce and resource-related challenges faced by rural clinics have been described,^50^ and COVID-19 created additional challenges, including care suspensions, staff redeployments, and staff turnover. Study staff also changed the delivery format of training and practice facilitation toward video-conference formats. Despite these challenges, most mailed FIT intervention components were successfully delivered by health plan vendors or clinic staff in this study (100% of the 1489 intervention-eligible enrollees were mailed an FIT, 88.5% were sent at least 1 advance notification, 78.1% were sent at least 1 reminder, and 57.9% of enrollees with an abnormal FIT result received some patient navigation for follow-up colonoscopy).

Notable changes to the health care context occurred prior to and during the study. CRC screening was an incentivized metric for Oregon’s Medicaid health plans from 2013 to 2019 but was not incentivized during the study interval. Accordingly, Oregon Medicaid CRC screening rates dropped by 8.3 percentage points (57.9% vs 49.3%) comparing 2019 to 2020 data.^51^ These factors likely led to lower prioritization of screening.^23^ Notably, 9.0% of enrollees in usual care clinics completed CRC screening within 12 months of eligibility determination, a much lower proportion than the 17% we previously report for Oregon Medicaid enrollees within the first 12 months of turning 50 years of age^8^ and lower than usual care groups in studies of community health centers (including our prior study, STOP CRC [14.5%],^40^ and SCORE [16.6%]^41^).

Our findings suggest that additional efforts may be needed to enhance the effectiveness of mailed outreach and patient navigation for rural Medicaid populations. Prior research has shown higher FIT return rates when visually appealing mailers and electronic trackers are used.^52^ Future evaluations may consider (1) combining mailed outreach with telehealth-delivered educational visits, (2) addressing social drivers of health, (3) providing health plan– or clinic-based incentives (or both), and (4) using patient-centered materials and approaches.^53,54^

Our study had notable strengths, including the focus on rural Medicaid enrollees. Our prior collaboration with some participating practices likely facilitated implementation, despite challenges introduced by COVID-19.^23^ The number of included clinics allowed for greater generalization of findings than previous work. Because claims data were used for our primary outcome, variation in data quality across our small, rural clinics was minimized. Several analyses not reported here are planned for separate publications, including an analysis of year 2 program delivery (clinic level) and repeat FIT completion (individual level) and a cross-ACCSIS consortium analysis of cost-effectiveness.

### Limitations

This study has limitations. Claims data may undercapture screening events, especially for individuals who disenroll in Medicaid, although disenrollment was found to be relatively low (4.4% were disenrolled within 6 months) during our study interval, likely due to national policies from 2020 to 2023 allowing continued coverage for Medicaid-enrolled individuals without showing proof of eligibility,^55^ leading to more complete capture of data than in prior Medicaid claims evaluations.^27^ Furthermore, our sensitivity analysis showed little impact on effectiveness estimates when disenrolled individuals were excluded from the analysis. We did not specifically assess the effects of advance notifications and reminders; however, this has been reported in prior research by our team.^43,44,56^ Finally, we experienced incomplete intervention delivery; advanced statistical methods may be needed to maintain study-group comparability to perform robust per-protocol analysis in implementation-effectiveness studies.

## Conclusions

In this cluster randomized clinical trial of rural clinics, mailed FIT outreach and patient navigation boosted participation in CRC screening among Medicaid enrollees. More efforts are needed to address low participation in both FIT testing and follow-up colonoscopy. Future research might explore how to reach individuals for preventive care who are enrolled in Medicaid but do not have an established primary care clinic.

## Notes

Supplement 1.
Trial Protocol

Supplement 2. **eTable 1.** Baseline Characteristics of Eligible Adults in Analysis Sample (N = 5614) **eTable 2.** Baseline Characteristics of Individuals Who Had No Evidence of Colorectal Cancer Screening and Disenrolled Within 6-Month and 12-Month Evaluation Intervals **eTable 3.** Cross-Tabulation of Rural-Urban Classifications Using Rural-Urban Commuting Area Codes and Office of Rural Health Definitions

Supplement 3.
Data Sharing Statement
