# Supplementary material for: Mailed Outreach and Patient Navigation for Colorectal Cancer Screening Among Rural Medicaid Enrollees: A Cluster Randomized Clinical Trial
Source: JAMA Netw Open. 2025 Mar 17;8(3):e250928. doi: 10.1001/jamanetworkopen.2025.0928 (PMC11915063; doi:10.1001/jamanetworkopen.2025.0928)
Supplement: Supplement 1. — Trial Protocol [file jamanetwopen-e250928-s001.pdf]

Contents

SMARTER CRC Protocol
SMARTER CRC Phase 2 Statistical Analysis Plan

OHSU Knight Cancer Institute

Cancer Prevention, Control and Epidemiology Protocol

Screening More patients for CRC through Adapting and Refining Targeted Evidence-based Interventions in Rural settings (SMARTER CRC)

Funding Agency: National Cancer Institute / Cancer Moonshot Initiative

Type of Award: UH3CA244298

Principal Investigators: Melinda M. Davis, PhD
3181 SW Sam Jackson Park Rd.
Portland, OR 97239
503-494-4365

Gloria Coronado, PhD
Kaiser Permanente Northwest
Portland, OR
503-335-2400

Final Protocol Date: 11/1/2019

Protocol Revision Dates: 1/13/2020
7/22/2020
11/2/2020
3/17/2021
6/3/2021
8/16/2022
5/15/2023
06/07/2023

Contents

1.0 ABSTRACT (required) 4
2.0 BACKGROUND / RATIONALE (required) 4

Return to Top

3.0 OBJECTIVES (required) 5
4.0 STUDY POPULATION (required) 5
5.0 INCLUSION/EXCLUSION CRITERIA (required) 6
6.0 METHODOLOGY (required) 7
7.0 STUDY PROCEDURES AND SCHEDULE OF EVENTS (required) 7
8.0 TIME LINE AND MILESTONES (required) 9
9.0 BIOSTATISTICAL CONSIDERATIONS (required) 11
10.0 ETHICAL AND REGULATORY REQUIREMENTS 12
10.1 Protocol Review (required) 12
10.2 Informed Consent (only if applicable) 12
10.3 Changes to Protocol (required) 12
10.4 Privacy, Confidentiality, and Data Security (required) 12
10.5 Maintenance of Records (required) 13
10.6 OHSU IRB Reporting of Unanticipated Problems and Adverse Events (only if applicable) 13
10.7 MedWatch Reporting (only if applicable) 13
10.8 OHSU Knight Cancer Institute Data and Safety Monitoring Plan (only if applicable) 13
10.9 Inclusion of Women, Minorities and Children (only if applicable) 13
10.10 Inclusion of Children (only if applicable) 14
11.0 REFERENCES (required) 14
12.0 APPENDIX I - TOXICITY CRITERIA (only if applicable) 16

Return to Top

1.0 ABSTRACT

This two-phase project is designed to achieve the Cancer Moonshot objectives by reducing the burden of CRC on the US population. Specifically, we aim to improve CRC screening rates, follow-up colonoscopy, and referral to care in rural Medicaid patients by implementing a direct mail fecal testing program with targeted outreach and patient navigation for follow-up colonoscopy. We leverage partnerships with the Oregon Rural Practice-based Research Network (ORPRN), Kaiser Northwest Center for Health Research, and Medicaid Health Plans and deliver training and implementation support to participating rural primary care clinics using practice facilitation. For the pragmatic trial, we anticipate working with 3 CCOs and 30 clinics reaching approximately 4,500 Medicaid patients. For the scale up trial, we anticipate partnering with 20 organizations to facilitate program implementation with 130 primary care clinics (reaching 17,000+ rural Medicaid patients). The mailed FIT and patient navigation interventions will be implemented as part of standard care by health system or clinic staff.

In Phase I (Year 01), we will conduct a milestone driven pilot to build the necessary infrastructure for a large-scale trial, including adapting the clinic-health plan-vendor supported direct mail program for rural Medicaid patients that have not established care and/or never been screened; conducting a pilot study testing the feasibility and acceptability of patient navigation to support follow-up colonoscopy following an abnormal fecal test; engaging Medicaid Health Plans and recruiting 30 primary care clinics located in rural and frontier counties in Oregon; and developing the training and support materials needed to implement a large-scale trial in these settings.

In Phase II (Years 02-05), we will test our intervention using a two-arm cluster randomized control trial in 30 rural primary care clinics using program training and practice facilitation to support implementation. Participating clinics will be randomized into two groups: Intervention and Usual Care. Randomization will be stratified on health system. As in the pilot, the intervention combines: (1) a clinic-health plan-vendor supported direct-mail fecal testing program with targeted outreach for patients who have never been screened or who have yet to establish care and (2) patient navigation for those who are referred for colonoscopy as either the primary screening or for follow-up from an abnormal fecal test. We will evaluate effectiveness, implementation, and maintenance of the intervention through quantitative and qualitative measures. Results from the trial will inform scale-up of the program through partnerships with 20 regional and national organizations that serve rural/frontier primary care clinics using webinars, train-the-trainer workshops and collaborative learning activities.

2.0 BACKGROUND / RATIONALE

Colorectal cancer (CRC) is the third-leading cause of cancer deaths in the United States[2] and the second leading cause among Oregonians.[3] CRC is 90% curable with timely detection and appropriate treatment of precancerous growths.[1] If not found until a patient is symptomatic, however, survival rates drop to 50%.[4] However, 1 in 3 age-eligible adults is not up-to-date for CRC screening.[5] Projections indicate that increasing CRC screening to 80% from current levels could prevent 277,000 cases and 203,000 deaths from the disease over the next 12 years.[6] Achieving this goal will require concerted efforts as approximately 25 million US adults aged 50–75 (33%) are not currently up-to-date.[7]

CRC screening rates are particularly low among adults in rural communities and sub-populations within these settings (e.g., Medicaid enrollees, Hispanic patients, Native Americans).[1,8-10] Rural areas cover 97% of the US land area and are home to approximately 60 million people. Within rural areas, frontier counties are the most remote and sparsely populated (having fewer than 7 people per square mile).[11] CRC incidence and mortality are disproportionately high among residents of rural regions;[9,12] disparities driven in part by differences in adherence to screening guidelines.[9,13] Medicaid enrollees

Return to Top

are a key underserved group in rural areas. In 2016, Medicaid provided health insurance and access to preventive health services to 82 million people, including 1 million in Oregon.[14] Medicaid covers nearly 1 in 4 rural resident under age 65 (24%).[15] This is important as Medicaid members aged 50-64 years have relatively low rates of CRC screening, as demonstrated in national data (47% for Medicaid vs. 60% for private/Medicare aged 50-64)[16] and Oregon data for CRC screening in newly age eligible patients (34.9% for Medicaid vs. 42.8% for private).[17] Medicaid members also display less favorable CRC outcomes compared to commercially insured adults.[18,19]

Interventions are needed to address disparities is CRC screening, follow-up, and treatment in rural Medicaid patients. While mailed FIT and patient navigation are effective methods of improving screening and follow-up, no existing program incorporates these strategies into a resource-efficient, sustainable program that can be broadly implemented in rural geographic regions. Our study, Screening More patients for CRC through Adapting and Refining Targeted Evidence-based Interventions in Rural settings (SMARTER CRC), supports implementation of a targeted, multilevel program that incorporates tailored outreach, direct mail and patient navigation to address CRC disparities in rural Medicaid patients. A primary component of these interventions will be supporting collaborations between clinics, Medicaid health plans operating as ACOs, and commercial vendors to optimize and sustain program components. Through the course of a pilot test, large-scale pragmatic trial, and scale-up study, we anticipate working with 30 regional and national organizations to facilitate the program's implementation with an estimated 130 rural primary care clinics (17,000+ rural Medicaid patients). We will assess drivers of program success at the patient-, clinic-, and community-levels. SMARTER CRC will produce an implementation guide and resources to support program spread across rural settings serving Medicaid enrollees and other underserved populations.

SMARTER CRC is a partnership between faculty and staff at the Oregon Rural Practice-based Research Network (ORPRN) at Oregon Health & Science University (OHSU) and at the Kaiser Permanente Northwest Center for Health Research. This study fills key evidence and implementation gaps and supports Biden's Cancer Moonshot objectives by providing a model for how to rapidly adapt and scale-up multilevel interventions through clinic-health plan partnerships to reduce the burden of CRC on the US population.

3.0 OBJECTIVES

Primary Objective:

Adapt, pilot, then test the implementation and scale-up of targeted direct mail and patient navigation programs.

4.0 STUDY POPULATION

The study team will recruit eligible CCOs and rural clinics, and we will work in turn with clinics to engage providers, staff, and patients. Our eligibility criteria for CCOs, clinics, clinics staff, patients and organizational partners are below.

CCOs/CCO staff: 1) serving a majority of counties that are predominantly rural based on 2010 RUCA Codes (Codes 4-10); 2) willing to participate in data collection activities (e.g., producing claims data, interviews).

Clinics: 1) Clinics will be eligible for the cluster randomization if there are 30 or more patients eligible for screening, 2) are classified as rural according to RUCA (Codes 4-10) or Oregon Office of Rural Health designations, 3) are served by CCOs agreeing to participate in the project; and 4) willing to implement the intervention into their clinic for the study.

Return to Top

Clinic Staff/Providers: 1) employed as a clinician or ancillary staff member in a participating clinic; 2) willing to participate in data collection activities (e.g., interviews, observation, surveys).

Patients: 1) attributed to participating clinic; and 2) are enrolled in Medicaid or Dual eligible; 3) eligible for CRC screening;

a. For the subset of patients that will be invited to participate in key informant interviews, a 5th eligibility criteria is consent to participate.

Exclusion criteria: Clinics are excluded if they have current or ongoing participating in other mailed fecal testing research projects in the Medicaid population. Patients are excluded if they are current for screening, have comorbid conditions that make patients poor candidates for screening based on clinical judgment (e.g., end-stage renal disease, enrollment in hospice), are not an established patient or for other reasons documented by the clinics.

Community or regional/organizational partners (includes endoscopy providers, community-based outreach workers, or leaders from regional or national organizations who participate in the pilot, pragmatic trial, or scale-up study) 1) involved in study activities (training, care delivery); 2) willing to participate in data collection activities (e.g., trainings, interviews, surveys).

For this project, we expect to recruit at least three CCOs and 33 Oregon clinics, which allows up to a 10% attrition rate to achieve our target of 30 rural primary care clinics. Based on Census Bureau data (https://www.census.gov/quickfacts/or), as of 2017, Oregon residents are 50.4% female and 13.1% Hispanic or Latino. Also based on Census data, Oregon resident diversity consists of 2.2% African American, 1.8% American Indian or Alaskan Native, 0.4% Native Hawaiian and other Pacific Islander, 4.7% Asian, 87.1% white, and 3.8% two or more races. Based on the Census Bureau's data, we expect the participating individuals to make up a representative sample of Medicaid individuals across the state.

5.0 INCLUSION/EXCLUSION CRITERIA

Inclusion criteria:

	Included	*Excluded*
Children		All patients we recruit will be at least 45 years of age or older, and clinic/CCO staff will be at least 18 years of age or older.
Elderly	Yes – we anticipate that a limited number of clinic and CCO staff, or community organization representatives may be elderly; we limit our patient recruitment to those aged 45-75.	
Rural	Yes	
Inner City	No	
Low Income	Yes	
Disabled	Yes	
Chronic Care	Yes	
End of Life	Yes - This is possible, but we predict limited numbers because of the types of individuals we are recruiting: clinic and CCO staff, and patients who are not currently in hospice care.	
Minorities	Yes	

Return to Top

Both Genders	Yes	

This study will not include any vulnerable populations. We will not collect any information about subjects' status as prisoners, pregnant women, children, neonates, and/or adults lacking capacity.

6.0 METHODOLOGY

Throughout this trial we will collect data from multiple sources to assess process and outcomes data and drivers of program success at the patient-, clinic-, and community-levels aligned with a social ecological model for the quality of cancer care. These sources of data include:

- CCO interview, readiness assessment, and survey
- Clinic survey, readiness assessment, observation and interviews
- Patient interviews
- Interviews and surveys with regional and organizational partners
- Patient outcomes data (e.g., claims, vendor reports and electronic health record) and patient navigation registry data

7.0 STUDY PROCEDURES AND SCHEDULE OF EVENTS

CCO interview, readiness assessment, and survey: CCOs that express interest in participating in the trial will be invited to participate in a semi-structured interview (in-person or via phone) and complete a baseline survey to confirm CCO characteristics (e.g., size, number of primary care clinics in rural/frontier counties) as well as information about CCO data infrastructure, relationships with clinics, and quality improvement capacity. Readiness questions will be drawn from the literature and explore the CCO's prior experience with payment and quality improvement initiatives related to CRC screening and follow-up care.

Clinic survey, readiness assessment, observation and interviews: For clinics expressing interest in the trial, we will conduct semi-structured interviews (in-person or via phone) with clinic leadership to assess clinic characteristics, capacity and readiness to adopt the program and participate in the trial. Clinic characteristics will include descriptive data (e.g., ownership, primary care clinician number and FTE, EHR vendor and version), baseline CRC screening rates and if the clinic currently utilizes audit and feedback, and details on current workflows related to CRC screening and follow-up (including provider CRC screening modality preferences). Capacity questions will explore general capacity as well as specific capacity related to CRC screening. Readiness questions will draw on prior work by Weiner, Hannon, and others,[20] and utilize a clinic-level readiness survey piloted by our team during Dr. Davis' K07.

We will conduct clinic surveys, interviews, and observations with clinic staff in various roles related to the program (e.g., outreach workers, patient navigators, quality improvement leads) to assess clinic/health system level factors that may influence outcomes. These assessments will happen at baseline and after implementation of the pilot as well as for each arm of the trial (post-implementation at 6-9 months as well as implementation context, maintenance, and sustainability approximately 12 months later [i.e., clinic exit interviews]).

Patient interviews: We will conduct one-on-one interviews with patients who receive the direct mail and patient navigation programs. Interviews will explore patient experiences with the program, including reaction, acceptability, satisfaction, and perceptions of usefulness; facilitators of, and barriers to, participating in the program and obtaining a follow-up colonoscopy; unintended consequences; and suggestions for improvement

Return to Top

Interviews and surveys with regional and organizational partners: To assess perceptions of the intervention and its impact on the broader community, we will interview CCO leaders, endoscopy providers (e.g., GI specialists, general surgeons, primary care clinicians) who treated study participants, and community organizations, including transportation service providers. Up to three interviews will occur throughout the duration of the study, including at baseline (pre-implementation), at the mid-point (approximately 12 months after the first-year implementation start), and post-implementation (approximately 12 months after the second-year implementation start). To evaluate the impact of scale-up activities we will conduct interviews and brief evaluation surveys with organizational leaders who participate in the trainings.

Patient outcomes data and patient navigation registry (Evaluation): Direct mail outreach and patient navigation activities will be tracked using reports from direct mail vendors, claims data from participating Medicaid health plans, clinic data from the electronic health record, chart abstraction data conducted by ORPRN or clinic staff, and data on navigation from a REDCap database. The mailed FIT and patient navigation interventions will be delivered by clinic and health plan staff as part of standard care. Clinics will be randomized to implement the interventions in different years to provide comparison groups. Claims data will be sent to the research team at OHSU from the participating payers based on their data pull of patients eligible for CRC screening in the clinics randomized to implement the intervention. Vendor reports will be sent to the CCO and research team based at OHSU. A clinic-level chart audit will be used to evaluate CRC screening and follow-up colonoscopy for patients with an abnormal FIT in intervention and control clinics.

Data for this project (i.e., process and survey data elements and patient navigation outcomes (such as number of patients who received patient navigation, number of patients with missed / canceled appointments or inadequate bowel preparation), will be stored in OCTRI's installation of REDCap, a highly secure and robust web-based research data collection and management system.

Features of REDCap that protect participants' privacy and data security include:

- Physical Security: OCTRI's REDCap software is housed on servers located in ITG's Advanced Computing Center providing locked physical security
- Electronic Security: The REDCap servers are housed behind both the OHSU firewall and a second ACC firewall. All transmissions of data from the application are encrypted over HTTPS with the industry standard TLS 1.1 protocol (AES 256-bit encryption).
- Controlled User Access: REDCap employs a robust multi-level security system that enables researchers to easily implement "minimum necessary" data access for their research staff, including specification of data fields that are identifiers. This feature includes “single click” ability to provide completely deidentified (removing all identified data fields and shifting dates) for analysis or other purposes. User activities are logged to enable auditing of all data access. Access is integrated with OHSU's network such that users who are also OHSU employees are authenticated against their OHSU network credentials.
- Data Integrity: REDCap is jointly managed in accordance with OHSU Information Security Directives by ACC staff and members of OCTRI's Biomedical Informatics Program, ensuring fidelity of database configuration and back-ups. User activities are logged to enable auditing of all data changes.

Data from the vendor and REDCap database will be merged with claims data to generate evaluation reports. To avoid bias in colonoscopy capture across baseline and follow-up time-points, ORPRN practice facilitators will perform a chart audit on all patients who were eligible for the direct mail program. The chart audit will confirm FIT completion and monitor for colonoscopy referral and receipt, pathology results, and referral to care (i.e. surveillance or cancer treatment), as indicated.

Researchers are requesting a waiver of authorization to access medical record data. ORPRN study staff plan to conduct the chart audit at each clinic over the course of 1-3 days. Data will be stored on the OHSU instance of REDCap or stored securely on OneDrive and ORPRN staff and select Kaiser Permanente research team members will have access to the data by utilizing a password-protected file on OneDrive or a REDCap user login. OneDrive and REDCap user permissions will be matched to

Return to Top

roles as described in the established Data Use Agreement between Kaiser Permanente and OHSU and to the IRB. PHI will never be disclosed outside of the study team and all PHI will be destroyed upon completion of the study. Through data use agreements, a limited dataset will be shared with the sponsor, the National Cancer Institute. The following data elements will be included in the limited data set: patient identifier, age group, sex, Hispanic or Latino origin, race, state of residence, county of residence and primary health insurance. When possible, the data set will also include primary language, ZIP code of residence, individual history of CRC, most recently performed screening test date and result, and diagnostic colonoscopy date and result.

SCHEDULE OF EVENTS

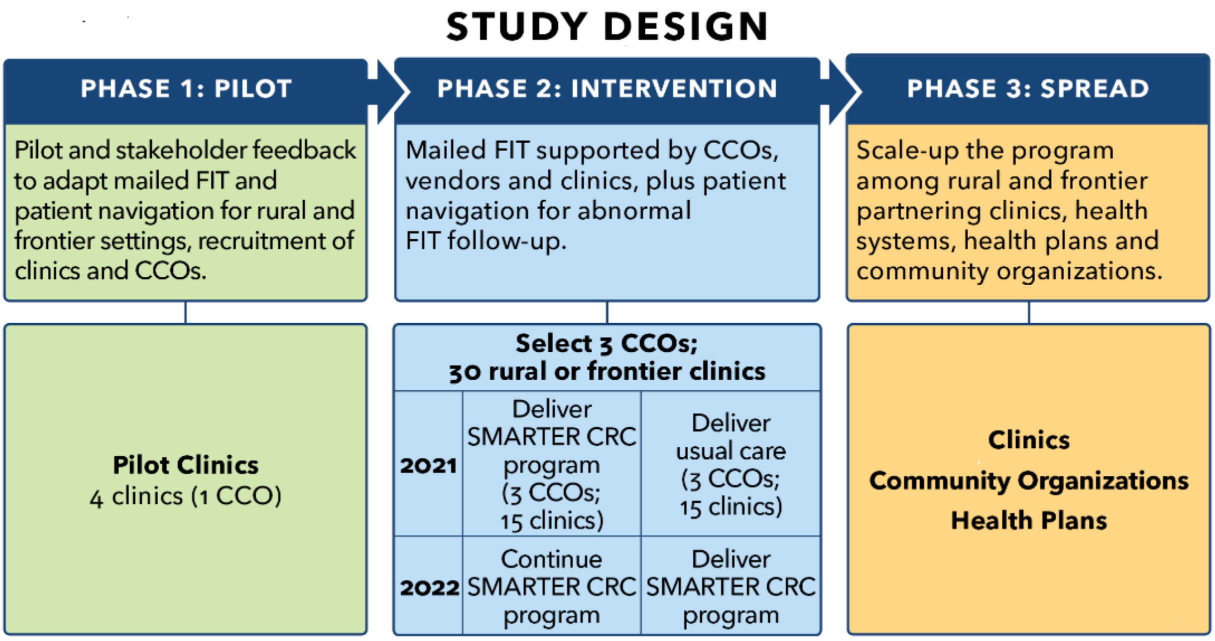

8.0 TIMELINE AND MILESTONES

Phase I practice and CCO participants will be recruited Fall 2019, with the pilot intervention complete by the end of year 1. Phase II practice and CCO participants will be recruited in Fall/Winter 2020-2021 and first year of implementation will occur in 2021, with second-year implementation occurring in Spring 2022.

Phase I Milestones and Timeline

Aim	Milestones	Information sources	Measurement / Validation	Timeline
**1.** Adapt direct mail materials and outreach to hard-to engage rural patients	**a.** Identify and recruit rural Medicaid patients to participate in Boot Camp Translation.	ORPRN, CHARA, CCOs, primary care clinics	Number approached and engaged. Demographic and geographic characteristics	Oct 2019 – Dec 2019
	**b.** Host Boot Camp Translation sessions; adapt program and identify outreach for patients at risk (unestablished, never screened).	Boot Camp Translation, relevant literature	Session attendance, evaluations, modifications of program messaging, timing, materials	Jan – May 2020
**2.** Conduct pilot to test the feasibility, effectiveness and acceptance of patient navigation program.	**a.** Update registry tools (tools will track total FIT kit mailings, test results, receipt of follow-up care, and display patient's previous CRC screening history). Implement patient navigation pilot.	Claims, vendor/lab data, EHR data<br><br>Navigation registry	Registry; vendor to identify patients needing follow-up; tools for tracking navigated patients and CRC-related outcomes in EHR or registry.	Oct 2019 – Apr 2020
	**b.** Obtain data on patients participating in direct mail program and FIT results; Assess the pilot intervention's preliminary reach and effectiveness	Claims, vendor/lab data, EHR data	N FITs completed / FITs mailed; across stratification variables.<br><br>N patients with abnormal FITs;	Apr – Jul 2020

Return to Top

	based on receipt of direct mail, FIT or colonoscopy completion, and proportion of patients receiving navigation.		documented receipt of navigation and outcome.	
	**c.** Assess the interventions feasibility and acceptance, based on one-on-one interviews with patients and debrief interviews with clinic/CCO staff.	Relevant literature	Feedback from patients and providers.	Apr – Jul 2020
**3.** Engage CCOs and recruit clinics; conduct baseline assessment; use results from pilot to prepare to conduct a large-scale, pragmatic implementation-effectiveness trial and scale-up (see Phase II).	**a.** Creation of a list of inclusion and exclusion criteria for CCOs and clinic participation in Phase II.	Relevant literature, statistical power requirements	Feedback from local advisory board on inclusion and exclusion criteria	Jan – Mar 2020
	**b.** Develop CCO and clinic recruitment materials	Relevant literature	Feedback from local advisory board	Jan – Mar 2020
	**b.** Successfully engage 3 CCOs and 30 rural/frontier clinics for the main trial	Feasibility data from pilot study	List of participating CCOs and selected clinic characteristics	Mar – Sep 2020
	**d.** Develop manual outlining trial protocols (including quality assurance protocols), scopes of work, and associated budgets.	Feasibility data from pilot study	Feedback from advisory board on recruitment materials; study protocol; scope of work	Apr – Sep 2020

Aims	Assessment/ Measurement	Data Sources	Description	Timeline
**Aim 1.** Conduct a large-scale pragmatic study, cluster randomized control trial, to assess the implementation, effectiveness, and maintenance of the program piloted in Phase I in 30 rural primary care clinics (n ~ 3,960 patients aged 45 – 75) using practice facilitation to support implementation. Using a mixed methods approach, identify patient-, clinic/health system-, and payer/policy/community-level factors that are associated with reach, effectiveness, implementation and maintenance and to assess program adaptations.	Assessment of effectiveness (patient and clinic level).	Administrative data, claims data, EHR data from clinics, vendor data, laboratory data, survey data.	Patient Level Outcome (Primary): Completion of any CRC screening<br><br>Clinic Level Outcome: Proportion of Medicaid patients screened for CRC<br><br>We will compare the intervention vs. control clinics after adjusting for baseline at 6 and 12 months. The primary endpoint is 6 months. Secondary outcomes: completion of testing types (fecal testing, FIT-DNA, CT Colonography, Colonoscopy, Flex Sigmoidoscopy; patient level) and % completion (clinic level); time to screening from study-eligible patient list pull (patient level); FIT results (patient level); follow-up colonoscopy completion (patient level); time to colonoscopy from abnormal FIT result (patient level); and adenomas or cancers detected (patient level).	2021-Sep 2024
	Assessment of implementation (compliance rate with program components at clinic level).	Administrative data, claims data, vendor data, laboratory data, survey data including debriefs.	Proportion of core activities performed (e.g., mailed FITs, patient navigation calls) by clinics/CCOs	2021-2024
	Assessment of maintenance (compliance rate with program over time at clinic level).	Administrative data, claims data, vendor data, laboratory data, survey data.	Proportion of intervention clinics sustaining program in second year (N core activities sustained in second year/ N core activities, in intervention clinics/CCOs). Effectiveness of the program in intervention clinics in Year 2.	2021-2024
	Assessment of maintenance (compliance rate with program at patient level)		Proportion of patients who completed FIT in Year 1 who complete in Year 2, in intervention clinics.	

Return to Top

	Assessment of program second year implementation and adaptations.	Clinic/CCO surveys and interviews.	Types of and reasons for program adaptations, based on Wiltsey-Stirman FRAME framework.	May 2023-Sep 2024
**Aim 2.** Partner with regional and national organizations (n~20) to scale-up the program to additional clinics serving rural and underserved patients in high priority geographic regions of the US (n ~ 130 clinics; 17,000+ patients) using webinars, train-the-trainer workshops and collaborative learning approaches. Assess trainings delivered, program adoption and adaptations and determinants of dissemination success.				
	**a.** Assessment of adoption by clinics and community organizations.	Participation in workshops, training, and collaborative learning activities; survey data; use of program tools and implementation materials.	N clinics, community organizations, and staff that participate in training workshops, train-the-trainer sessions, and collaborative learning activities.<br>Adoption as reported on 6-month survey data of workshop participants.<br>Use of program tools; downloads of training and implementation materials.	2021-2024
	**b.** Assessment of program adaptations.	Clinic and community organization surveys and interviews.	Types of and reasons for program adaptations.	Oct 2023-Sep 2024

9.0 BIOSTATISTICAL CONSIDERATIONS

Phase I: Because this is the pilot phase, in which the primary purpose is to examine the feasibility and acceptability of the intervention and not to test a hypothesis, we have not performed a power analysis to determine the required sample size for this phase. Instead our primary goal was to evaluate the feasibility and acceptability of the program.[21-23]

Phase II, Aim 1 (Implementation-Effectiveness Trial): We will test our hypotheses using a two-arm cluster randomized control trial. Participating clinics will be randomized into two groups: Intervention and Usual Care. Randomization will be stratified on health system. Our study design aligns with the core principles of the PRECIS-2 framework.[24-26] Our **eligibility criteria** are broad and consistent with the realities of rural primary care (e.g., small sample sizes, limited quality improvement capacity). Our patient recruitment approach for the direct mail and navigation programs is aligned with routine care and standard quality improvement approaches and requires no individual consent. Participating clinics will serve populations that display CRC screening disparities, and be diverse in terms of EHR, rural geographic locations, and populations served. Our intervention is flexible and is designed to be adapted according to payer and clinics' resources and preferences. Our primary outcome, completion of any CRC screening, is pragmatic and relevant to patients, clinics, and payers (CCOs), and our primary analysis follows the intention-to-treat concept.[27] We will collect data 6 times from the CCOs. Data will be transferred every 6 months from the Spring of 2021 to the Fall of 2023 to be able to capture up to 18 months of follow-up following each mailing.

The primary effectiveness outcome of this study at the patient level is the completion of any colorectal cancer (CRC) screening (for study-eligible patients). Consistent with the pragmatic nature of the trial, we will use claims, vendor data, and EHR data for calculating CRC screening outcomes. Comparing the likelihood of receiving CRC screening between intervention and usual care groups at 6-month post participant list-pull date, we hypothesize that CRC screening will be more likely in patients allocated to the intervention group versus the usual care group. To examine the effectiveness of the program CRC screening completion at 6 months, we will use the generalized form of the

Return to Top

hierarchical linear model (HLM; using a logit link and binomial distribution, aka multilevel logistic regression) to account for the clustering of patients within clinics and the assignment to arm at the clinic level. The variables that will be included in the model are the clinic baseline screening rate (if available) and a binary indicator of arm (1=tailored, 0=standard) as fixed effects and clinic is a random effect. A positive and significant coefficient for arm would provide support for the effectiveness of the intervention. We will do a moderator analysis at all levels to explore the impact of patient and clinics characteristics (see outcomes table above), and implementation components affecting outreach on completion likelihood in groups of patients determined at analysis (e.g. never screened, Hispanic, etc.).

10.0 ETHICAL AND REGULATORY REQUIREMENTS

10.1 Protocol Review

The protocol and informed consent form for this study must be reviewed and approved in writing by the OHSU Knight Cancer Institute (Knight) Clinical Research Review Committee (CRRC) and appropriate Institutional Review Board (IRB) prior to any patient being registered on this study.

10.2 Informed Consent

We are requesting a waiver of documentation of written consent for participation in the main trial. The waiver of informed consent is requested because CRC screening is standard care and all activities proposed to be undertaken with the research project are minimal risk. There are no interventions or other procedures for participants for which written documentation of consent is normally required for research activities.

We intend to collect verbal consent from participants who participate in qualitative interviews. Researchers will disseminate an information sheet for the surveys and interviews and will obtain verbal consent from participants before continuing with the study interview. Completion of the survey will serve as consent to participate. We will obtain verbal consent prior to the interview.

The research team will conduct chart review for all participants who are eligible for CRC screening. The high number of charts to be reviewed renders contact of each individual to obtain written authorization impractical as contact information may not be available and the time and resources it will require to obtain written consent is not commensurate with a low-risk chart audit review. A Waiver of Authorization for the chart review components of this study is included with the application. All data collected via chart audit will be handled per section 10.4.

10.3 Changes to Protocol

Any modification of this protocol must be documented in the form of a protocol revision or amendment signed by the principal investigator and approved by the CRRC and IRB, before the revision or amendment may be implemented. The only circumstance in which the amendment may be initiated without regulatory approval is for a change necessary to eliminate an apparent and immediate hazard to the patient. In that event, the investigator must notify the CRRC and IRB in writing within 5 working days after the implementation. Investigators holding the IND must notify FDA of substantive changes to the protocol.

10.4 Privacy, Confidentiality, and Data Security

This research study involves minimal risk to human subjects. The magnitude of harm or discomfort anticipated in the proposed research is not greater in and of itself than that ordinarily encountered in everyday life. The information sheet will describe the project purpose, study activities, participant’s rights, benefits, and who to contact with questions and will be used to obtain consent.

Data entered or sent to the study offices will be handled in a highly confidential manner consistent with the high standards established at OHSU and ORPRN. Data presented in all presentations and publications will not be associated with the name of any participating person or practice. All computer systems at OHSU and ORPRN are protected from possible external access using network security systems. Only study researchers and staff at ORPRN and Kaiser Permanente will have access to the data. We will use industry standard Secure Sockets Layer (SSL) technology with server and client certificates to insure the confidentiality of data use and any data transfer.

Return to Top

Surveys and interview transcripts will be de-identified; unique numerical identifiers will be assigned for clinic and practice participants. The audio recordings from interviews will be destroyed after analysis. Text files will be stored in a password protected, encrypted computer file on a secure workstation at OHSU.

10.4a. Risks and Benefits

- Risks to Subjects

There is minimal foreseeable risk, discomfort, hazard, or inconvenience to the subjects relating to this chart review. There is a minimal risk of breach of confidentiality. The unlikely event of a loss of confidentiality could occur through a data transfer oversite.

- Potential Benefits to Subjects

There are no direct benefits to subjects.

10.5 Maintenance of Records

Study data will be stored on OHSU's secure Box.com.

If the investigator relocates or for any reason withdraws from the study, the study records will be transferred to OHSU Knight Cancer Institute Clinical Research Management. Records will be maintained according to sponsor requirements.

OHSU is one of multiple study sites funded by the National Cancer Institute (NCI) through this funding opportunity called the Accelerating Colorectal Cancer Screening and Follow-up through Implementation Science (ACCSIS). NCI is establishing a data use agreement with each ACCSIS grantee for submission of a limited consolidated data set in order for NCI to establish a repository for further evaluation of funded activities.

10.6 OHSU IRB Reporting of Unanticipated Problems and Adverse Events

Unanticipated Problems (UP) and Adverse Events (AE) will be reported to IRB according to the policies, procedures and guidelines posted on the OHSU IRB web site:

- Fatal and life-threatening UP will be reported to OHSU IRB within 5 days of notification of the event. All other UP reports will also be submitted to OHSU IRB no later than 5 days of occurrence or notification of the event. Copies of the report documents will be kept in the study regulatory binder.
- UP and AE reports are submitted through OHSU eIRB and will be reviewed by OHSU Knight Cancer Institute and IRB. Monthly accumulative reports will be reviewed by a DSMC Oncologist and forwarded to the CRRC.

10.7 MedWatch Reporting

Not applicable.

10.8 OHSU Knight Cancer Institute Data and Safety Monitoring Plan

Not applicable. Patients will not be treated under this protocol.

10.9 Inclusion of Women, Minorities and Children

The projected gender, racial, and ethnic composition of the study will represent that of the state of Oregon.

Table 1: Population Demographics - Oregon (%)

Ethnic Category	Sex/Gender		
	Females	Males	Total
Hispanic or Latino	5.85	5.85	11.7
Not Hispanic or Latino	44.15	44.15	88.3

Return to Top

**Ethnic Category: Total of all subjects***	50	50	100*
**Racial Category**			
American Indian or Alaskan Native	0-1	0-1	1.4
Asian	1.85	1.85	3.7
Black or African American	0-1	0-1	1.8
Native Hawaiian or other Pacific Islander	0-1	0-1	0.3
White	41.8	41.8	83.6
More than one race	1.9	1.9	3.8
Unknown/Other	2.65	2.65	5.3
**Racial Category: Total of all subjects***	50	50	100*
TOTALS	50.4	49.6	100*

Source: U.S. Census Bureau, 2010 *Totals may not equal 100 due to rounding.

Table 2: Projected Accrual for the Present Study

**Ethnic Category**	**Sex/Gender**			
	Females	Males	Unknown	Total
Hispanic or Latino	1,159	1,141	-	2,299
Not Hispanic or Latino	7,756	7,633	-	15,389
Unknown	-	-	-	-
**Ethnic Category: Total of all subjects***	8,915	8,774	-	17,688
**Racial Category**				
American Indian or Alaskan Native	178	176	-	354
Asian	356	351	-	708
Black or African American	178	176	-	354
Native Hawaiian or other Pacific Islander	90	87	-	177
White	7,756	7,632	-	15,389
More than one race	356	351	-	708
Unknown	-	-	-	-
**Racial Category: Total of all subjects***	8,915	8,774	-	17,688*

Source: Adapted from U.S. Census Bureau, 2010 *Totals may not equal 100 due to rounding.

10.10 Inclusion of Children

This protocol does not include children for the following reason: We are surveying and interviewing adult staff working in primary care practices and CCOs. Patients who are interviewed are in the CRC screening-eligible population (50-75 years old).

11.0 REFERENCES

1. American Cancer Society. *Colorectal Cancer Facts & Figures 2017-2019.* Atlanta, GA2017.
2. Cronin KA, Lake AJ, Scott S, et al. Annual Report to the Nation on the Status of Cancer, part I: National cancer statistics. *Cancer.* 2018;124(13):2785-2800.

Return to Top

3. American Cancer Society. Oregon Cancer Statistics At a Glance. 2018; https://cancerstatisticscenter.cancer.org/#!/state/Oregon. Accessed Jan 25, 2019.
4. Wiegering A, Ackermann S, Riegel J, et al. Improved survival of patients with colon cancer detected by screening colonoscopy. *International journal of colorectal disease.* 2016;31(5):1039-1045.
5. Siegel RL, Miller KD, Fedewa SA, et al. Colorectal cancer statistics, 2017. *CA: A Cancer Journal for Clinicians.* 2017;67(3):177-193.
6. Fedewa SA, Ma J, Sauer AG, et al. How many individuals will need to be screened to increase colorectal cancer screening prevalence to 80% by 2018? *Cancer.* 2015;121(23):4258-4265.
7. Centers for Disease Control and Prevention. *Morbidity and Mortality Weekly Report.* Atlanta, GA 01/27/2012 2012. 3.
8. Sabatino SA, White MC, Thompson TD, Klabunde CN. Cancer screening test use - United States, 2013. *MMWR Morbidity and mortality weekly report.* 2015;64(17):464-468.
9. Henley SJ, Anderson RN, Thomas CC, Massetti GM, Peaker B, Richardson LC. Invasive Cancer Incidence, 2004-2013, and Deaths, 2006-2015, in Nonmetropolitan and Metropolitan Counties - United States. *Morbidity and mortality weekly report Surveillance summaries (Washington, DC : 2002).* 2017;66(14):1-13.
10. Cole AM, Jackson JE, Doescher M. Urban-rural disparities in colorectal cancer screening: cross-sectional analysis of 1998-2005 data from the Centers for Disease Control's Behavioral Risk Factor Surveillance Study. *Cancer medicine.* 2012;1(3):350-356.
11. Rural Health Information Hub. Health and Healthcare in Frontier Areas. 2018; https://www.ruralhealthinfo.org/topics/frontier#how-much. Accessed Jan 25, 2019.
12. Blake KD, Moss JL, Gaysynsky A, Srinivasan S, Croyle RT. Making the Case for Investment in Rural Cancer Control: An Analysis of Rural Cancer Incidence, Mortality, and Funding Trends. *Cancer epidemiology, biomarkers & prevention : a publication of the American Association for Cancer Research, cosponsored by the American Society of Preventive Oncology.* 2017;26(7):992-997.
13. Lansdorp-Vogelaar I, Kuntz KM, Knudsen AB, van Ballegooijen M, Zauber AG, Jemal A. Contribution of screening and survival differences to racial disparities in colorectal cancer rates. *Cancer epidemiology, biomarkers & prevention : a publication of the American Association for Cancer Research, cosponsored by the American Society of Preventive Oncology.* 2012;21(5):728-736.
14. Commission TMaCPaA. MACStats: Medicaid and CHIP Data Book. 2017; The Medicaid and CHIP Payment and Access Commission. Accessed October 11, 2018.
15. Foutz J, Artiga S, Garfield R. *The Role of Medicaid in Rural America.* Menlo Park, CA: The Henry J. Kaiser Family Foundation;2017.
16. de Moor JS, Cohen RA, Shapiro JA, et al. Colorectal cancer screening in the United States: Trends from 2008 to 2015 and variation by health insurance coverage. *Preventive medicine.* 2018;112:199-206.
17. Davis MM, Renfro S, Pham R, et al. Geographic and population-level disparities in colorectal cancer testing: A multilevel analysis of Medicaid and commercial claims data. *Preventive medicine.* 2017;101:44-52.
18. Fitzgerald T, Lea, C. , Atluri, P. , Brinkley, J., Zervos, E. . Insurance Payer Status and Race Explains Much of the Variability in Colorectal Cancer Survival. . *Journal of Cancer Therapy.* 2014;5:1223-1233.
19. Andrew AS, Parker S, Anderson JC, et al. Risk Factors for Diagnosis of Colorectal Cancer at a Late Stage: a Population-Based Study. *Journal of general internal medicine.* 2018;33(12):2100-2105.
20. Coronado GD, Sos C, Talbot J, Do HH, Taylor VM. To be healthy and to live long, we have to exercise: psychosocial factors related to physical activity among Cambodian Americans. *Journal of community health.* 2011;36(3):381-388.
21. Coronado GD, Thompson B, Tejeda S, Godina R. Attitudes and beliefs among Mexican Americans about type 2 diabetes. *J Health Care Poor Underserved.* 2004;15(4):576-588.
22. Coronado GD, Taylor VM, Hislop TG, et al. Opinions from ESL instructors and students about curricula on hepatitis B for use in immigrant communities. *J Cancer Educ.* 2008;23(3):161-166.

Return to Top

23. Coronado GD, O'Connell MA, Anderson J, Loest H, Ogaz D, Thompson B. Undergraduate cancer training program for underrepresented students: findings from a minority institution/cancer center partnership. *J Cancer Educ.* 2010;25(1):32-35.
24. Kraemer HC, Mintz J, Noda A, Tinklenberg J, Yesavage JA. Caution regarding the use of pilot studies to guide power calculations for study proposals. *Archives of general psychiatry.* 2006;63(5):484-489.
25. Oregon ECHO Network. Connect and Learn. 2018. Accessed Jan 24, 2019.
26. Proctor E, Silmere H, Raghavan R, et al. Outcomes for implementation research: conceptual distinctions, measurement challenges, and research agenda. *Administration and policy in mental health.* 2011;38(2):65-76.
27. Weiner BJ. A theory of organizational readiness for change. *Implementation science : IS.* 2009;4:67.

12.0 APPENDIX I - TOXICITY CRITERIA

Not applicable.

Phase 2 Statistical Analysis Plan (SAP) for Screening More patients for CRC through Adapting and Refining Targeted Evidence-based Interventions in Rural settings (SMARTER CRC)

NCI trial identifier	NCI-2021-01032
ClinicalTrials.gov identifier	NCT04890054
SAP version	Final, deidentified for publication
SAP version date	8/13/2024
NCI grant number	1UG3CA244298
IRB of record	Oregon Health & Science University

SAP revision history

Version edited	Date edited	Section number changed	Description and reason for change
5/12/2021	8/9/2022	2.1.1	Replaced "days from FIT completion" with "days from abnormal FIT result" in the "time to colonoscopy" secondary outcome measure. This aligns with prior research.<br><br>Replaced "brand" with "type" in the observed rate of abnormal fecal test results. This allows comparisons based on stool-test characteristics and not commercial brand.<br><br>Removed "How many enrollees with a cancer detected were referred to cancer care, and what was the time to referral?" as we were unable to collect data for referral to cancer care.
		5.2	Added note to inclusion criteria that age will be lowered [from 50] to 45 once state Medicaid pays for testing. During Year 1, the age recommendation had not yet been lowered to 45, so our minimum age was 50. In Year 2, the age had been lowered so our minimum age was 45.

Return to Top

			Added "including dually eligible for Medicaid and Medicare" to inclusion criteria. This is pragmatic and consistent with prior literature.
		7.1.3	Age and sex were added as individual level covariates to all analyses because of their known relationship to the outcomes.
8/9/2022	8/13/2024	3.2.2	Table with randomization strata and names of CCOs and clinics removed for deidentification.
		3.6	Table with dates of list pull, FITs mailed, and chart audits completed removed for deidentification.

Roles

**Role**	**Name**
Trial statistician	Robert Durr, MPH
Trial statistician	Maryan Carbuccia Abbott, MS, MPP
Trial statistician	Jean Hiebert Larson, MS
Senior statistician	Michael C. Leo, PhD
Senior statistician	Katrina L. Ramsey, MPH
Principal investigator	Gloria Coronado, PhD
Principal investigator	Melinda M. Davis, PhD
Site investigator	Amanda F. Petrik, PhD
Project manager	Anna C. Edelmann, MScN

Return to Top

Contents

1. Introduction 20
2. Background 20
    2.1. Rationale and research questions 20
        2.1.1. Secondary questions 20
    2.2. Hypotheses 20
    2.3. Abbreviations 20
3. Study materials 20
    3.1. Trial design 20
    3.2. Randomization 21
        3.2.1. Definition of clinic as randomization unit 21
        3.2.2. Definition of strata for randomization: 21
        3.2.3. Randomization 21
    3.3. Sample size 21
    3.4. Interim analyses 21
        3.4.1. Planned sample size adjustment 21
        3.4.2. Stopping rules 21
    3.5. Timing of final analysis 21
    3.6. Timing of outcome assessments 21
4. Statistical principles 22
    4.1. Levels of confidence and p values 22
        4.1.1. Adjustment for multiplicity 22
    4.2. Adherence and protocol deviations 22
        4.2.1. Definition and assessment of adherence 22
        4.2.2. Presentation of adherence 22
        4.2.3. Contamination 22
    4.3. Analysis population 22
        4.3.1. Eligible Clinics 22
        4.3.2. Eligible patients 22
        4.3.3. Intervention arm patients 22
5. Summary of Study Population 22
    5.1. Clinic eligibility 22
        5.1.1. Inclusion 22
        5.1.2. Exclusion 22
    5.2. Patient eligibility 22
        5.2.1. Inclusion 22
        5.2.2. Exclusion 23
    5.3. Withdrawal/follow-up 23
    5.4. Baseline characteristics 23
        5.4.1. Sources 23
6. Outcome definitions 27

Return to Top

6.1. Definitions of time frames 27
6.2. Primary outcome 27
6.3. Exploratory outcome(s) 29
6.4. Descriptive outcomes 29
6.4.1. Clinic-level descriptive outcomes 30
6.4.2. CCO-level descriptive outcomes 30
7. Analysis 30
7.1. Analysis methods 30
7.1.1. Graphic analysis 30
7.1.2. Primary outcome 32
7.1.3. Covariate adjustment 32
7.1.4. Assumption checking 33
7.1.5. Alternative methods if distributional assumptions not met 33
7.1.6. Sensitivity analyses 33
7.2. Subgroups/moderator analysis 33
7.2.1. Definitions 33
7.2.2. Approach 34
7.3. Missing data 34
7.4. Additional analyses 34
7.5. Harms 34
7.6. Statistical software 34
8. References 35
8.1. Non-standard statistical methods 35
8.2. Statistical packages 35
8.3. Trial documents 35

Introduction

This statistical analysis plan provides guidelines for the final presentation and analysis for the SMARTER CRC Trial.

This study collects information to provide a model for how to rapidly adapt and scale-up multilevel interventions through clinic-health plan partnerships to reduce the burden of colorectal cancer (CRC) on the United States population. This study may improve CRC screening rates, follow-up colonoscopy, and referral to care in rural Medicaid enrolees.

Background

Rationale and research questions

SMARTER CRC aims to test the implementation, effectiveness, and maintenance of a mailed fecal test and patient navigation program to improve rates of CRC screening, follow-up colonoscopy, and referral to care in clinics serving rural Medicaid enrolees.

Our primary effectiveness outcome is receipt of any CRC screening within 6 months of enrolee identification. Our primary implementation outcome is health plan- and clinic-level rates of program delivery, by component (mailed FIT and patient navigation).

Secondary questions

1. Does time to CRC screening differ among enrolees in clinics allocated to intervention vs. usual care?
2. How do fecal test completion proportions at 6 months and 12 months differ among enrolees in clinics allocated to intervention vs. usual care?
3. What is the observed rate of abnormal fecal test results, overall and by fecal test type (FIT, FIT-DNA)?
4. What proportion of study enrolees, by intervention condition, with an abnormal fecal test result were appropriately referred to follow-up colonoscopy?
5. What proportion of study enrolees, by intervention condition, with an abnormal fecal test result appropriately received a follow-up colonoscopy within 12 months of abnormal test result?
6. Does time to follow-up colonoscopy differ (up to 12 months) among enrolees in clinics allocated intervention vs. usual care?
7. What were the findings on colonoscopy among study enrolees (i.e., n adenoma detected, n advanced adenoma detected, n cancer detected as a proportion of colonoscopies completed)?
8. What intervention components were delivered by CCOs and clinic staff (scrub list, mailed FIT, pre-FIT notifications, post-FIT reminders, patient navigation) and to what proportion of ‘eligible’ enrolees?

Hypotheses

We hypothesize that a higher proportion of enrolees in clinics allocated to the intervention vs. usual care will complete CRC screening within 6 months of eligibility identification (claims list pull date).

Abbreviations

CCO	Coordinated Care Organization
FIT	Fecal immunochemical test
FOBT	Fecal occult blood test
MOP	Manual of operations
SAP	Statistical Analysis Plan

Study materials

Trial design

This is a parallel cluster-randomized trial (Year 1) with continued implementation in intervention clinics and delayed implementation in usual care clinics (Year 2) with clinics allocated 1:1 to mailed fecal test (FIT) and patient navigation or usual care. A cluster-randomized design was chosen because it could

Return to Top

minimize the potential for contamination (versus individual-randomized designs) and could minimize bias due to changes in secular trends (versus stepped-wedge designs).

Clinics randomly allocated to the intervention in Year 1 receive a list of eligible enrolees from the coordinated care organizations (CCO), clinic staff review the list and select patients to receive mailing based on pre-determined criteria (clinic staff were trained in list scrubbing). The CCO mails FITs to enrolees on the scrubbed lists and the clinics/CCOs deliver pre-FIT notification and/or reminders to support FIT completion (e.g., phone calls, text messages). Clinic staff trained in patient navigation assist enrolees in arranging follow-up testing after abnormal screening results.

Randomization

Definition of clinic as randomization unit

Clinic recruitment resulted in an initial list of 33 recruited clinics. Beginning with this list, we created three small clusters of clinics (two pairs and one trio) within the same health system to serve as randomization units. These clinics had common staff or enrolees, lacked claims data at the level of individual clinics, or were too small to otherwise meet eligibility criteria. This resulted in 29 clinic units for randomization.

Definition of strata for randomization:

Ten randomization strata, each with n=2 to n=6 clinic units, were formed based on health care organization affiliation. Multiple randomization units from the same organization (see above) were treated as a stratum; lone randomization units within each CCO were grouped into one stratum for individual randomization units with a single participating location and, where applicable, one stratum for clinics operating as individual randomization units with more than one participating location.

[Table with randomization strata and names of CCOs and clinics removed for deidentification]

Randomization

Units were randomized 1:1 into intervention and control groups. Randomization was stratified on health system designation (e.g. hospital-affiliated clinics or independent clinics) and performed by the project statistician in two batches in April 2021 (2 CCOs) and May 2021 (1 CCO), with timing based on data availability. Each batch consisted of five strata, with 2-6 clinic units within a stratum. The randomized allocation sequence was generated using STATA version 16 (College Station, TX).

Sample size

Reported p-values are based on the corresponding adjusted odds ratio that account for clustering and covariates. All analyses were two-tailed and considered significant if $p<.05$. Power calculations were derived using PASS 15, and showed that a 10 percentage-point change in screening rates could be detected, assuming 106 patients per clinic, an ICC of .03, and a baseline screening rate of 44.6%.

Interim analyses

None planned.

Planned sample size adjustment

None.

Stopping rules

None.

Timing of final analysis

Year 1 data will be analyzed after the dataset is finalized.

Year 2 data will be analyzed separately.

Timing of outcome assessments

Patient-level outcomes are related to the normal delivery of preventive health care and will mainly be collected using health care claims, with some additional information from chart reviews and mailed FIT vendor reports (for CCO 3 only). Claims are requested approximately every six months but are cumulative, i.e. every request uses the same start date for the search window, with the end date being (approximately) the date when the query is executed. This strategy is intended to allow for fixing errors and to protect against data loss in administrative databases. The last such request occurs 18 months after the initial list pull, when claims are expected to be largely complete.

Return to Top

[Table with dates of list pull, FITs mailed, and chart audits completed removed for deidentification]

Statistical principles

Levels of confidence and p values

Statistical hypothesis testing will be two-sided at the .05 level of significance. Report 95% confidence intervals.

Adjustment for multiplicity

None planned.

Adherence and protocol deviations

Definition and assessment of adherence

Adherence is not relevant in this study, as any clinics that do not wish to participate withdraw from the study.

Presentation of adherence

None.

Contamination

Some patients who were scrubbed from the initial eligibility list or from usual care clinics may receive mailed FIT kits because of eligibility through other programs (i.e. mailed outreach to Medicare enrolees).

Analysis population

Eligible Clinics

The intent-to-treat population of eligible clinics will include all clinics that were enrolled and randomized into a study arm.

Eligible patients

The intent-to-treat population of eligible patients will include all CCO enrolees who were identified as eligible for CRC screening by the CCO, before review or "scrubbing" by clinic staff or elimination of bad addresses.

Intervention arm patients

The analysis set of intervention arm patients (eligible patients – see definition above) will include the subset of eligible patients who are assigned to intervention arm clinics in Year 1.

Summary of Study Population

Clinic eligibility

The number of recruited clinics eligible and how many were excluded due to violating each inclusion/exclusion criterion will be tabulated.

Inclusion

- >=30 patients eligible for screening
- Located in a ZIP code classified as rural using either RUCA or Oregon Office of Rural Health codes
- Served by one of the participating CCOs

Exclusion

- Current or ongoing participation in other mailed fecal testing research projects

Patient eligibility

Inclusion

- Age-eligible for CRC screening: Aged 50-74 (age to be lowered to 45 once state Medicaid pays for testing)
- Enrolled in Medicaid (including dually eligible for Medicaid and Medicare)
- Not current for screening at the time that the list was generated, i.e. no evidence of
    - colonoscopy within 10 years
    - flexible sigmoidoscopy or CT colonography in the past 5 years
    - FIT within 12 months
    - FIT-DNA in the past 36 months

Return to Top

Exclusion

- Current for screening (see above)
- Other medical reason that screening is not appropriate (i.e., colorectal cancer, colorectal disease, hospice/end-of life care, etc.)

Withdrawal/follow-up

Timing of and reason(s) for clinic withdrawal from follow-up will be presented in the CONSORT diagram. Patients cannot withdraw from the study. However, they can lose coverage. Counts of patients and the timing relative to list pull will be tabulated.

Baseline characteristics

Sources

Table A. List of baseline data

Data source	Characteristics	Definition
**Clinic-level variables**		
Clinic Intake Survey, coded by research team	Federal designation	▪ Rural Health Clinic<br>▪ Federally-Qualified Health Center (FQHC)<br>▪ Tribal Health Center<br>▪ No Federal Designation
Clinic Intake Survey, coded by research team	Clinic network structure	*See Table B.*
CCO	CCO	CCO that handles clinic's claims.
CCO	Eligible patients per clinic	Less than 100<br>100 to 200<br>200 or more
**Patient-level variables**		
CCO original list pull<br>*medicaid_id*	Patient Medicaid ID	Provided by CCO. Identifier used to merge datasets.
CCO original list pull<br>*firstname*	Patient first name	Provided by CCO. Identifier used to merge datasets.
CCO original list pull<br>*lastname*	Patient last name	Provided by CCO. Identifier used to merge datasets.
CCO original list pull<br>*dob*	Patient date of birth	Provided by CCO. Identifier used to merge datasets.
accsis_id	Unique research ID	Assigned by research team.
redcap_id	REDCap database ID	Assigned by REDCap database. Used to merge CCO data and REDCap data.
study_year	Cohort assignment	All patients in this SAP should have *study_year* = 1.
CCO original list pull	CCO that submitted patient	▪ CCO 1<br>▪ CCO 2<br>▪ CCO 3
cl_accsis_id	Unique clinic identifier	Assigned by research team.
CCO original list pull<br>*clinic*	Patient clinic assignment	Clinic 1 - 29
intervention	Indicator for study group, determined by randomization assignment	▪ 1 = intervention<br>▪ 0 = usual care
Itt	Indicator for intent-to-treat (ITT) population, from original list pull	▪ 1 = include in ITT analyses (includes "randomized in error")<br>▪ 0 = non-ITT; include only in intervention analyses

Return to Top

why_not_itt	Description of reason for decision to remove patient from ITT sample	*Qualitative, not used in analysis.*
why_not_itt_dt	Date when patient was determined to be removed from ITT sample	*Qualitative, not used in analysis.*
CCO original list pull<br>*listpull_dt*	List pull date, provided by CCO	Date
CCO original list pull<br>*age*	Patient age	Calculated as "age as of list-pull date" using date of birth and list pull date
CCO original list pull<br>*age_group*	Patient age group, calculated	▪ 50-54<br>▪ 55-59<br>▪ 60-64<br>▪ 65-74
CCO original list pull<br>*gender*	Patient gender (where missing in original list pull, obtained from next follow-up dataset with non-missing value)	▪ F = female<br>▪ M = male
CCO original list pull<br>*race*	Patient race (where missing in original list pull, obtained from next follow-up dataset with non-missing value)	*Note that CCO-reported category "Asian and Pacific Islander" is recoded to "Asian."*<br>▪ American Indian or Alaskan Native<br>▪ Asian<br>▪ Black<br>▪ Native Hawaiian or Other Pacific Islander<br>▪ White<br>▪ Other<br>▪ Two or more races<br>▪ Missing
race_cat	Simplified race categories	▪ White = White<br>▪ Non-white, other = non-white and non-missing<br>▪ Unknown/not reported = missing
CCO original list pull<br>*hispanic*	Patient Hispanic ethnicity (where missing in original list pull, obtained from next follow-up dataset with non-missing value)	▪ Hispanic<br>▪ Not Hispanic<br>▪ Missing
CCO original list pull<br>*language*	Patient language (where missing in original list pull, obtained from next follow-up dataset with non-missing value)	List of 20+ languages
language_cat	Simplified language categories	▪ English (includes English-Spanish with "English" listed first)<br>▪ Spanish (includes English-Spanish with "Spanish" listed first)<br>▪ Other/Unknown (includes Arabic, Cambodian, Cantonese, Chinese, Falam, Korean, Persian, Punjabi, Russian, Vietnamese, missing)
CCO original list pull<br>*state*	Patient state of residence, provided by CCO	• Oregon<br>• Other
CCO original list pull<br>*zip*	Patient ZIP Code of residence, provided by CCO	Identifier used to merge datasets.
CCO original list pull, Oregon Office of Rural Health (ORH)	Patient county of residence, calculated by merging zip codes	*Note that this is missing (county = "not in Oregon") for non-Oregon patients.*

Return to Top

county	and Office of Rural Health (ORH) county information.	
CCO original list pull, Oregon ORH<br>*rurality_orh*	ORH rurality designation, calculated by merging *zip* and ORH designations.	*Note that this is missing for non-Oregon patients.*<br>▪ Urban<br>▪ Rural<br>▪ Frontier<br>▪ Missing
CCO original list pull, RUCA<br>*rurality_ruca*	RUCA rurality designation, calculated by merging *zip* and RUCA designations.	Numeric 1-10, or missing.
ruca_group	Simplified RUCA groups, calculated using *rurality_ruca*.	▪ Metropolitan = RUCA codes 1, 2, 3<br>▪ Micropolitan = RUCA codes 4, 5, 6<br>▪ Small town = RUCA codes 7, 8, 9<br>▪ Rural = RUCA code 10<br>▪ missing = missing RUCA code
CCO original list pull, 2nd follow-up data (CCO 3 only)<br>*insurance*	Insurance status, provided by CCO; For CCO 3, insurance designation was obtained from 2nd follow-up data pull.	• Medicaid<br>• Medicare-Medicaid
CCO follow-up data<br>*coverage_start_dt*	Date patient insurance coverage started, provided by CCO	Date
CCO follow-up data<br>*coverage_end_dt*	Date patient insurance coverage terminated, provided by CCO; Where more than one date was provided, the latest date was retained.	Date
CCO original list pull, follow-up data, claims, vendor data (CCO 3 only)<br>*priorscreen_yn*	Prior CRC screening	*See Table B.*
CCO original list pull, follow-up data, claims, vendor data (CCO 3 only)<br>*priorfit_yn*	Prior FIT screening	*See Table B.*
CCO 3rd follow-up data<br>*pcp_2021*	Number of visits during mailing year (2021), provided by CCO	▪ None<br>▪ 1<br>▪ 2-5<br>▪ 6+<br>▪ unknown

Return to Top

Table B. Definitions for baseline data

Variable	Category	Definition
**Clinic-level variables**		
Clinic network structure	Individual clinic	Clinic is operating as a single location and is not affiliated with specialty care
	Clinic with multiple locations	Clinic is associated with other primary care clinic sites but is not affiliated with specialty care or centralized ownership
	Hospital affiliated clinic	Clinic is owned / operated by a hospital system. These clinics may still report that they are not part of a "system."
	Health care network affiliated clinic	Clinic is owned / operated by a larger external entity that provides specialty services (e.g., orthopedics, surgery) in addition to primary care. This entity owns clinics/provides services across multiple regions.
**Patient-level variables**		
Prior CRC screening	Prior CRC screening	*priorscreen_yn* = 1 if there is evidence of any CRC screening occurring before the list-pull date in the original CCO datasets, follow-up data ("most recent screening" and also claims), or vendor data. Eligible screening types include FIT/FOBT, colonoscopy, FIT-DNA, CT colonography, and flexible sigmoidoscopy.
	No prior screening	*priorscreen_yn* = 0 if there is no evidence of any CRC screening before the list-pull date.
Prior FIT screening	Prior FIT screening	*priorfit_yn* = 1 if there is evidence of any FIT or FOBT screening occurring before the list-pull date in the original CCO datasets, follow-up data ("most recent screening" and also claims), or vendor data.
	No prior FIT screening	*priorfit_yn* = 0 if there is no evidence of any FIT or FOBT screening before the list-pull date.

Return to Top

Outcome definitions

Definitions of time frames

Time intervals measured in months or years will end on the same day of the month *N* number of months after the index date, inclusive.

Primary outcome

We will evaluate intervention effectiveness by assessing whether enrolees in the intervention clinics are more likely to obtain any CRC screening compared to patients in the usual care clinics within 6 months of the date the enrolees were identified as eligible (claims list pull date) (Table 1).

Completion of 1 or more types of CRC screening as recorded by CCO (claims) and/or mailed FIT vendor. Types of screening and medical billing codes are detailed in Table C below. Successful *FIT screening* is defined as having a claim for a resulted FIT kit.

Other colorectal cancer screening is defined as one of the following codes in claims:

Table C1. Colorectal cancer screening codes

Category	CPT/HCPCS Procedure Code(s)	Notes
**FOBT/FIT**	82270, 82274, G0328	CRC screening == 1<br>FIT screening == 1
**Flexible Sigmoidoscopy**	45330, 45331, 45332, 45333, 45334, 45335, 45337, 45338, 45339, 45340, 45341, 45342, 45345, 45346, 45347, 45349, 45350, G0104	CRC screening == 1<br>FIT screening == 0
**Colonoscopy**	44388, 44389, 44390, 44391, 44392, 44393, 44394, 44397, 44401, 44402, 44403, 44404, 44405, 44406, 44407, 44408, 45355, 45378, 45379, 45380, 45381, 45382, 45383, 45384, 45385, 45386, 45387, 45388, 45389, 45390, 45391, 45392, 45393, 45398, G0105, G0121	CRC screening == 1<br>FIT screening == 0
**CT Colonography**	74261, 74262, 74263	CRC screening == 1<br>FIT screening == 0
**FIT-DNA**	81528, G0464	CRC screening == 1<br>FIT screening == 0

Table C2. List of CRC screening variables

Data source	Characteristics	Definition
**Baseline variables**		
CCO original list pull, follow-up data, claims, vendor data (CCO 3 only)<br>*priorscreen_yn*	Prior CRC screening	*See Table B.*

Return to Top

CCO original list pull, follow-up data, claims, vendor data (CCO 3 only)<br>*priorfit_yn*	Prior FIT screening	*See Table B.*
**Outcome variables**		
CCO original list pull, follow-up data, claims, vendor data (CCO 3 only)<br>*crcscreen_6m_yn*	CRC screening within 6 months of list-pull date	Calculated =1 *if any is true* within window (list-pull date to list-pull date + 183 days):<br>▪ Most recent screening variables in member-level list indicate CRC screening within window<br>▪ Claim for screening (see Table B) within window.<br>▪ Vendor data indicates FIT returned within window.
CCO original list pull, follow-up data, claims, vendor data (CCO 3 only)<br>*fitscreen_6m_yn*	FIT screening within 6 months of list-pull date	Calculated =1 *if*:<br>▪ crcscreen_6m_yn = 1 *and*<br>▪ crc_type == FIT or FOBT
CCO original list pull, follow-up data, claims, vendor data (CCO 3 only)<br>*crcscreen_12m_yn*	CRC screening within 1 year of list-pull date	Calculated =1 *if any is true* within window (list-pull date to list-pull date + 366 days):<br>▪ Most recent screening variables in member-level list indicate CRC screening within window<br>▪ Claim for screening (see Table B) within window.<br>▪ Vendor data indicates FIT returned within window.
CCO original list pull, follow-up data, claims, vendor data (CCO 3 only)<br>*fitscreen_12m_yn*	FIT screening within 1 year of list-pull date	Calculated =1 *if*:<br>▪ crcscreen_12m_yn = 1 *and*<br>▪ crc_type == FIT or FOBT

Time frame: Up to 6 (and 12 months) after initial list was pulled

Level of analysis: Individual patient

Return to Top

Chart review

To obtain data for outcomes that were unavailable in claims data (FIT result, etc.), research team members (e.g., practice facilitators, data analysts) conducted chart audits of enrolees who were identified as having completed CRC screening (based on claims data, vendor data, and REDCap data). These research team members gathered FIT results and, for those with abnormal FIT results, colonoscopy outcomes (colonoscopy date, presence of adenomas, cancer). For practical reasons, these research team members were unblinded to study allocation and worked directly with clinic staff to gather data from EHR records.

Secondary outcomes

Additional effectiveness outcomes include receipt of FIT within 6 months and 12 months, and FIT result; and among those with an abnormal FIT result, follow-up colonoscopy receipt within 12 months, and time to colonoscopy. We also will assess colonoscopy outcomes (e.g. n adenomas, cancers detected), and referral to cancer care among those with cancer detected.

Table D. Secondary patient-level outcomes

Outcome	Definition	Denominator (if applicable)	Time frame
Completion of FIT screening and FIT result	Successful FIT screening is defined as having a kit that was returned and successfully processed for a screening result. Kits that were returned incorrectly do not count as successful cancer screenings.	ITT eligible patients	Up to 6 months after initial list pull
Time to colorectal cancer screening	Time (days) to completion of any type of colorectal cancer screening (lab processing date for at-home tests)	ITT eligible patients	Up to 6 months after initial list pull

Table E. Secondary clinic-level outcomes

Outcome	Definition	Denominator (if applicable)	Time frame
Proportion of eligible patients who complete CRC screening	See 0"Primary outcome" above for definition. Aggregate at clinic level.	ITT eligible patients by assigned clinic	Up to 6 months after initial list pull

Exploratory outcome(s)

Completed colonoscopy | eligible for follow-up colonoscopy

Completions of diagnostic follow-up, usually colonoscopy, among enrolees with an abnormal screening result. This is an exploratory outcome. The hypothesis is that patient navigation will lead to more complete follow-up after abnormal stool-test results.

Time frame: Up to 6 months after screening (lab processing date or date in clinic, depending on screening type)

Level of analysis: Individual patient

Descriptive outcomes

Table F. Patient-level descriptive outcomes

Numerator	Definition	Denominator	Time frame

Return to Top

Numerator	Definition	Denominator	Time frame
Completion of screening, type:<br>Fecal testing (1/0)<br>FIT-DNA (1/0)<br>CT colonography (1/0)<br>Colonoscopy (1/0)<br>Flexible sigmoidoscopy (1/0)	See 0"Primary outcome" above for definitions. Exclude diagnostic workups after screening.	Eligible patients from list pull (ITT population)<br>patients who are screened (what share of screenings is each type)	Up to 6 months from list pull
FIT results<br>positive<br>negative<br>not returned<br>returned but faulty<br>not sent (error)	Result as reported by vendor or from chart review	Patients from intervention clinics who completed FIT kits	Up to 6 months from list pull
Received at least one live phone contact from Navigator (1/0) from REDCap		Enrolees in intervention clinics with an abnormal FIT result	Up to 12 months from list pull
Follow-up colonoscopy (1/0) from claims and chart audit		Screen positive from chart review and vendor reports	Up to 12 months from list pull
Time to colonoscopy from abnormal fit result (days) from claims and chart audit	Compare time to FIT and time to follow-up colonoscopy, we will use Cox proportional hazards regression with shared frailty to account for the clustering of patients nested within clinics.	Abnormal fit from vendor reports and chart reviews	Up to 6 months, starting from abnormal test date
Adenoma or colorectal cancer detected by colonoscopy from chart audit (1/0)		Screened from claims and vendor reports<br>i.e. Pr(Diagnosis|Screening)	Up to 12 months from list pull

Clinic-level descriptive outcomes

Table G. Clinic-level descriptive outcomes

Numerator	Denominator	Time frame
Rate of referral to colonoscopy from chart audit	Patients with abnormal test results	Up to 12 months from list pull
Proportion scrubbed from eligible patient list with reasons from REDCap	Eligible patients from list pull in Mailed FIT arm	NA

CCO-level descriptive outcomes

Table H. CCO-level descriptive outcomes

Numerator	Denominator	Time frame
Rate of CRC screening from claims and vendor reports	Eligible patients from list pull	Up to 12 months from list pull
Rate of referral to colonoscopy from chart audit	Patients with abnormal test results	Up to 12 months from list pull

Analysis

Analysis methods

Graphic analysis

- For binary outcomes, summarize proportion at the clinic level and **plot** these proportions

Return to Top

    - separated by intervention/usual care with N on the x axis, where N is the eligible population at that clinic. Include a reference line for the arm-specific mean proportion (each clinic weighted equally) and the confidence interval for that proportion given the eligible population.
    - As a dot/box plot by study arm
- Kaplan-Meier curve of screening times by intervention arm
    - By clinic / CCO / study arm
    - With reminder call timing overlaid or adjacent

Return to Top

Primary outcome

To examine the effectiveness of the program on CRC screening completion at 6 months, we will use the generalized form of the hierarchical linear model (HLM; using a logit link and binomial distribution, aka multilevel logistic regression) to account for the clustering of patients within clinics and the assignment to arm at the clinic level. There is no longitudinal data, as everyone at baseline would not have been screened. The first level of the model will contain the covariates of age and sex and an intercept for person. Variability at the individual (patient) level (ε_{ij}) is modelled with a mean of 0 and variance $\pi^2/3$. The second level of the model (clinic) will include a binary indicator of arm (1=tailored, 0=standard) and two indicators for CCO (three CCOs dummy coded, randomization will serve as the reference group) as fixed effects and clinic as a random effect (μ_j). A positive and significant coefficient for arm would provide support for the effectiveness of the intervention. From this model, we will derive the estimated marginal proportions and associated 95% confidence intervals, as well as perform contrasts between the differences in proportions and their associated 95% confidence intervals. These estimates can be interpreted as the adjusted (for the covariates and nesting of patients within clinics) risks and risk differences.

Covariate adjustment

CCO (stratification variable) as fixed effect

Covariates are selected before unblinding the dataset and are correlated with the outcome. Including covariates that are correlated with the outcome can improve precision and reduce bias in the estimate of treatment effect (see Pocock, S. J., Assmann, S. E., Enos, L. E., & Kasten, L. E. (2002)). Subgroup analysis, covariate adjustment and baseline comparisons in clinical trial reporting: current practice and problems. Statistics in medicine, 21(19), 2917-2930). It is not practical to set a threshold for the correlation, but we will be looking for effects that correspond to a correlation of 0.3 or greater, because smaller effects are unlikely to improve the estimate of treatment effect.

A literature review is one step in identifying covariates (see table below).

Table I. List of covariates for analysis

Variable	Previously observed association with screening	Evidence (95% CIs)
CCO	No observed association	No current evidence
Gender	Females have greater response to mailed FIT outreach	FIT completion rates are higher in females vs. males: OR: 1.12 (,99, 1,28) Nielson et al. 2019
Age	Older patients have greater response to mailed FIT outreach	FIT completion rates are higher in adults >65 vs. <= 65: OR 1.42 95% CI (1.19, 1.69)

1: Nielson, C. M., Vollmer, W. M., Petrik, A. F., Keast, E. M., Green, B. B., & Coronado, G. D. (2019). Factors affecting adherence in a pragmatic trial of annual fecal immunochemical testing for colorectal cancer. *Journal of general internal medicine*, *34*, 978-985.

2: Singal, A. G., Gupta, S., Tiro, J. A., Skinner, C. S., McCallister, K., Sanders, J. M., ... & Halm, E. A. (2016). Outreach invitations for FIT and colonoscopy improve colorectal cancer screening rates: a randomized controlled trial in a safety-net health system. *Cancer*, *122*(3), 456-463.

3: Levy, B. T., Xu, Y., Daly, J. M., & Ely, J. W. (2013). A randomized controlled trial to improve colon cancer screening in rural family medicine: an Iowa Research Network (IRENE) study. *The Journal of the American Board of Family Medicine*, *26*(5), 486-497.

4: Goshgarian, G., Sorourdi, C., May, F. P., Vangala, S., Meshkat, S., Roh, L., ... & Croymans, D. M. (2022). Effect of patient portal messaging before mailing fecal immunochemical test kit on colorectal cancer screening rates: a randomized clinical trial. *JAMA Network Open*, *5*(2), e2146863-e2146863.

Return to Top

5: Brenner, A. T., Rhode, J., Yang, J. Y., Baker, D., Drechsel, R., Plescia, M., ... & Wheeler, S. B. (2018). Comparative effectiveness of mailed reminders with and without fecal immunochemical tests for M edicaid beneficiaries at a large county health department: A randomized controlled trial. *Cancer*, *124*(16), 3346-3354.
6: Hendren, S., Winters, P., Humiston, S., Idris, A., Li, S. X., Ford, P., ... & Fiscella, K. (2014). Randomized, controlled trial of a multimodal intervention to improve cancer screening rates in a safety-net primary care practice. *Journal of general internal medicine*, *29*, 41-49.

Table J. Covariates considered and rejected

Variable	Reason for not including as covariate
Ethnicity (Hispanic vs non-Hispanic white)	(expected low prevalence/high missingness)
Dual eligibility<br>(Medicaid and Medicare vs Medicaid only)	(expected low prevalence)
CRC screening history (yes vs never screened)	uncertainty about how well we can ascertain this history from CCO data
Established care (vs no visit in the past year)	uncertainty about how well we can ascertain this history from CCO data

Assumption checking

N/A

Alternative methods if distributional assumptions not met

Sensitivity analyses

We will also report (descriptively) the proportion of enrolees who were mailed a FIT that completed one within the 12-month evaluation interval. We will report the proportion of enrolees who received at least some navigation who completed a follow-up colonoscopy.

Subgroups/moderator analysis

Definitions

We will perform a moderator analysis of patient characteristics. Specifically:

Table K. Moderator list

Variable	Coding	Hypothesized difference in effect	Previous evidence
Dual eligible	Medicare and Medicaid = 1<br>Medicaid only = 0	dual eligible will be less responsive to intervention	FIT completion proportions were 18.8 – 22.4% among Medicaid enrollees and 15.9 – 18.9% in dual eligible enrollees (no test or interaction was performed)<br>Coronado et al. 2020
Rurality	OHA and RUCA codes	enrollees living in ZIP codes with frontier designations will be less responsive than all else (rural and urban)	7: population density <=1000 (rural) OR 1.45 vs 1.32 for more density, p=.26 for interaction
Sex	male vs female	females will be less responsive to the intervention than women	2: no interaction effect<br>5: males greater effect (RR 2.06 vs 1.47 for females), NS<br>7: 1.33 for F vs 1.42 for

Return to Top

			male, p=.33 for interaction
Prior FIT testing	Yes vs. No	Enrollees with no prior FIT testing will be less responsive	Prior FIT completion associated with 3-fold higher repeat FIT completion; OR 3.0 95% CI: 2.58, 3.47. Nielson et al. 2019

2: Singal, A. G., Gupta, S., Tiro, J. A., Skinner, C. S., McCallister, K., Sanders, J. M., ... & Halm, E. A. (2016). Outreach invitations for FIT and colonoscopy improve colorectal cancer screening rates: a randomized controlled trial in a safety-net health system. *Cancer*, *122*(3), 456-463.
5: Brenner, A. T., Rhode, J., Yang, J. Y., Baker, D., Drechsel, R., Plescia, M., ... & Wheeler, S. B. (2018). Comparative effectiveness of mailed reminders with and without fecal immunochemical tests for Medicaid beneficiaries at a large county health department: A randomized controlled trial. *Cancer*, *124*(16), 3346-3354.
7: O'Connor, E.A., Vollmer, W.M., Petrik, A.F. et al. Moderators of the effectiveness of an intervention to increase colorectal cancer screening through mailed fecal immunochemical test kits: results from a pragmatic randomized trial. Trials 21, 91 (2020). https://doi.org/10.1186/s13063-019-4027-7

Table L. Moderators considered but not tested

Variable	Reason for not testing
CCO	level 3 predictor better suited for CCM
Federal designation	level 3 predictor better suited for CCM
FIT type	level 2 or 3 predictor better suited for CCM
established care	(cannot evaluate with CCO data)
Hispanic ethnicity	(low prevalence high missingness expected)<br>(less responsive)
CRC screening history	(unable to assess with CCO data) less responsive (some measurement uncertainty)
Age	

Approach

We will determine whether there are moderator (aka heterogeneity of treatment effects, effect modification) effects based on the individual level characteristics of dual eligibility, rurality, sex, and prior fit testing. To statistically test for moderation, we will add the moderator to the first level of the model and cross-level product of the moderator and arm using the framework described in the analysis of the primary outcome. A significant coefficient for the cross-level product term would be evidence for moderation. We will also derive the estimated marginal means and associated 95% CIs within each subgroup by arm (i.e., simple main effects) and graph them to facilitate interpretation. We will conduct these analyses separately by moderator.

Missing data

No imputations, missing data will be treated as missing

Additional analyses

None

Harms

None

Statistical software

We will use Stata 17.0 to carry out the inferential analyses.

Return to Top

References

Non-standard statistical methods

Statistical packages

Trial documents

[Data Management Plan]

[Trial Primary File and Statistical Primary File]

[Study Protocol]

Based on:

Gamble, C., Krishan, A., Stocken, D., Lewis, S., Juszczak, E., Doré, C., Williamson, P. R., Altman, D. G., Montgomery, A., Lim, P., Berlin, J., Senn, S., Day, S., Barbachano, Y., & Loder, E. (2017). Guidelines for the content of statistical analysis plans in clinical trials. JAMA, 318(23), 2337–2343. https://doi.org/10.1001/jama.2017.18556

https://clinicaltrials.gov/ProvidedDocs/25/NCT02445625/SAP_000.pdf

Figure A

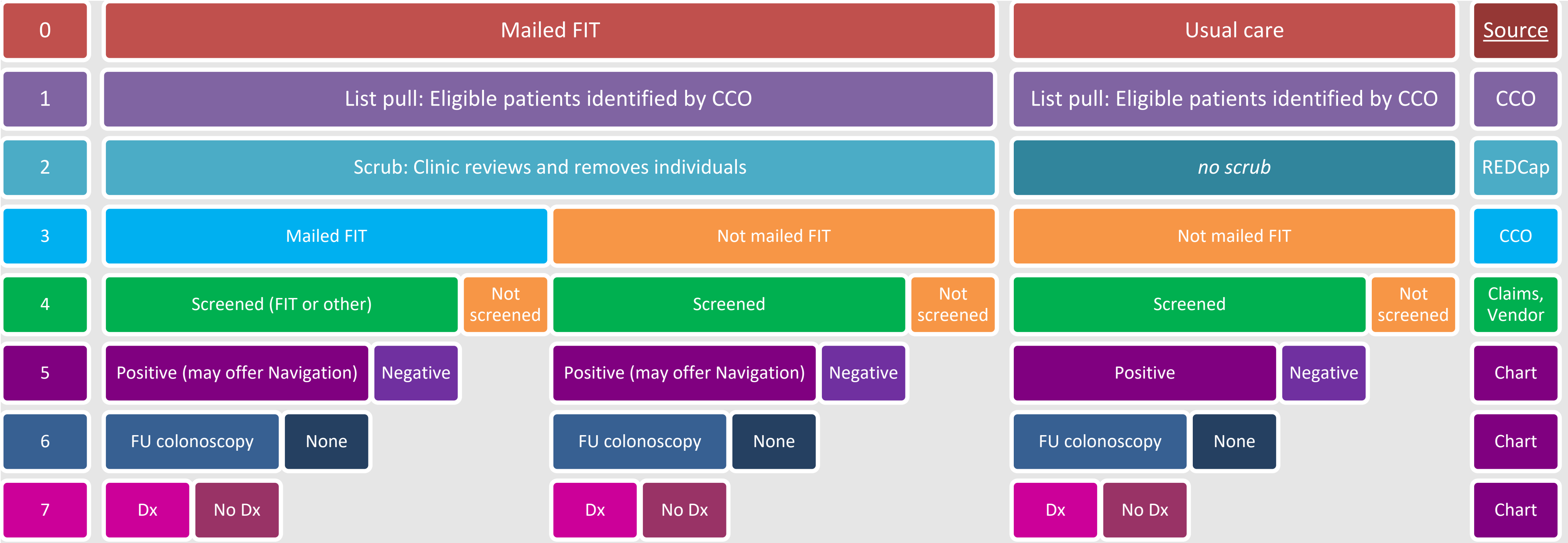
0
Mailed FIT
Usual care
Source
1
List pull: Eligible patients identified by CCO
List pull: Eligible patients identified by CCO
CCO
2
Scrub: Clinic reviews and removes individuals
no scrub
REDCap
3
Mailed FIT
Not mailed FIT
Not mailed FIT
CCO
4
Screened (FIT or other)
Not screened
Screened
Not screened
Screened
Not screened
Claims, Vendor
5
Positive (may offer Navigation)
Negative
Positive (may offer Navigation)
Negative
Positive
Negative
Chart
6
FU colonoscopy
None
FU colonoscopy
None
FU colonoscopy
None
Chart
7
Dx
No Dx
Dx
No Dx
Dx
No Dx
Chart
