# Supplementary material for: Mailed Outreach and Patient Navigation for Colorectal Cancer Screening Among Rural Medicaid Enrollees: A Cluster Randomized Clinical Trial
Source: JAMA Netw Open. 2025 Mar 17;8(3):e250928. doi: 10.1001/jamanetworkopen.2025.0928 (PMC11915063; doi:10.1001/jamanetworkopen.2025.0928)
Supplement: Supplement 2. — eTable 1. Baseline Characteristics of Eligible Adults in Analysis Sample (N = 5614) eTable 2. Baseline Characteristics of Individuals Who Had No Evidence of Colorectal Cancer Screening and Disenrolled Within 6-Month and 12-Month Evaluation Intervals eTable 3. Cross-Tabulation of Rural-Urban Classifications Using Rural-Urban Commuting Area Codes and Office of Rural Health Definitions [file jamanetwopen-e250928-s002.pdf]

Supplemental Online Content

Coronado GD, Petrik AF, Leo MC, et al. Mailed outreach and patient navigation for colorectal cancer screening among rural Medicaid enrollees: a cluster randomized clinical trial. *JAMA Netw Open*. 2025;8(3):e250928. doi:10.1001/jamanetworkopen.2025.0928

eTable 1. Baseline Characteristics of Eligible Adults in Analysis Sample (N = 5614)

eTable 2. Baseline Characteristics of Individuals Who Had No Evidence of Colorectal Cancer Screening and Disenrolled Within 6-Month and 12-Month Evaluation Intervals

eTable 3. Cross-Tabulation of Rural-Urban Classifications Using Rural-Urban Commuting Area Codes and Office of Rural Health Definitions

This supplemental material has been provided by the authors to give readers additional information about their work.

© 2025 Coronado GD et al. *JAMA Network Open*.

eTable 1. Baseline Characteristics of Eligible Adults in Analysis Sample (N = 5614)

Characteristic	All enrollees N=5,614	Intervention enrollees N = 2,613
	N (%)	N (%)
**Age**		
50-54	1,657 (29.5)	785(30.0)
55-59	1,698 (30.3)	812 (31.1)
60-64	1,585 (28.2)	726 (27.8)
65-74	674 (12.0)	290 (11.1)
**Sex**		
Female	2,948 (52.5)	1,334 (51.1)
Male	2,666 (47.5)	1,279 (48.9)
**Hispanic Ethnicity**	325 (6.2)	135 (5.2)
**Preferred Language**		
English	5,184 (92.4)	2,354 (90.1)
Spanish	204 (3.6)	82 (3.1)
Other/Unknown	226 (4.0)	177 (6.8)
**Race**		
Multi-racial /races other than White[a]	313 (5.6)	188 (7.2)
White	3,774 (67.2)	1,745 (66.8)
Unknown/not reported	1,527 (27.2)	680 (26.0)
**Rurality, Office of Rural Health Classifications**		
Urban	175 (3.1)	37 (1.4)
Rural	4,457 (79.4)	2,121 (81.2)
Frontier	950 (16.9)	439 (16.8)
Missing/Not in Oregon	32 (0.6)	16 (0.6)
**Insurance Status**		
Medicaid	4,555 (81.1)	2,157 (82.6)
Dual Medicaid- Medicare	1,059 (18.9)	456 (17.5)
**Number of Visits in 2021**		
None	2,205 (39.3)	1,065 (40.8)
1	673 (12.0)	299 (11.4)
2-5	1,724 (30.7)	794 (30.4)
6 +	1,012 (18.0)	455 (17.4)
**Ever had Prior Colorectal Cancer Screening** [b]	1,012 (18.0)	522 (20.0)

[a] Includes 170 American Indian or Alaska Native, 54 Asian, 36 Black, 6 Native Hawaiian or other Pacific Islander, 77 other, and 2 two or more races; race and ethnicity determined using administrative claims data.
[b] Includes 395 in intervention clinics and 464 in usual care clinics with prior FIT completion

© 2025 Coronado GD et al. *JAMA Network Open*.

eTable 2. Baseline Characteristics of Individuals Who Had No Evidence of Colorectal Cancer Screening and Disenrolled Within 6-Month and 12-Month Evaluation Intervals

Characteristic	N enrollees	Disenrolled within 6 months	Disenrolled within 12 months
		N= 246<br>N (row %)	N = 421<br>N (row %)
**Overall**	5,614	246 (4.4)	421 (7.5)
**Age**			
50-54	1,657	66 (4.0)	104 (6.3)
55-59	1,698	87 (5.1)	146 (8.6)
60-64	1,585	55 (3.5)	105 (6.6)
65-74	674	38 (5.6)	66 (9.8)
**Sex**			
Female	2,948	100 (3.4)	192 (6.5)
Male	2,666	146 (5.5)	229 (8.6)
**Hispanic Ethnicity**	325	8 (2.5)	9 (2.8)
**Preferred Language**			
English	5,184	238 (4.6)	408 (7.9)
Spanish	204	2 (1.0)	3 (1.4)
Other/Unknown	226	6 (2.8)	10 (4.7)
**Race**			
Multi-racial /races other than White[a]	313	18 (5.8)	28 (9.0)
White	3,774	161 (4.3)	274 (7.3)
Unknown/not reported	1,527	67 (4.4)	119 (7.8)
**Rurality, Office of Rural Health Classifications**			
Urban	175	7 (4.0)	12 (6.7)
Rural	4,457	199 (4.5)	341 (7.7)
Frontier	950	36 (3.8)	61 (6.4)
Missing/Not in Oregon	32	4 (12.5)	7 (21.9)
**Insurance Status**			
Medicaid	4,555	214 (4.7)	370 (8.1)
Dual Medicaid-Medicare	1,059	32 (3.0)	51 (4.8)
**Number of Visits in 2021**			
None	2,205	135 (6.1)	213 (9.61)
1	673	27 (4.0)	44 (6.5)
2-5	1,724	60 (3.5)	111 (6.4)
6 +	1,012	24 (2.4)	54 (5.3)

© 2025 Coronado GD et al. *JAMA Network Open*.

**Ever had Prior Colorectal Cancer Screening [b]**	1,012	34 (3.4)	61 (6.0)
**Study Condition**			
Intervention	2,613	132 (5.1)	202 (7.7)
Usual care	3,001	114 (3.8)	219 (7.3)

[a] Includes 170 American Indian or Alaska Native, 54 Asian, 36 Black, 6 Native Hawaiian or other Pacific Islander, 77 other, and 2 two or more races; race and ethnicity determined using administrative claims data.

[b] Includes 395 in intervention clinics and 464 in usual care clinics with prior FIT completion

© 2025 Coronado GD et al. *JAMA Network Open*.

eTable 3. Cross-Tabulation of Rural-Urban Classifications Using Rural-Urban Commuting Area Codes and Office of Rural Health Definitions

	Office of Rural Health (n=5,614)*		
	Urban, n	Rural, n	Frontier, n
**Urban/rural status (RUCA)**			
Metropolitan (RUCA code 1-3)	133	652	0
Micropolitan (RUCA codes 4-6)	42	3092	265
Small Town (RUCA codes 7-9)	0	260	323
Rural Areas (RUCA code 10)	0	453	362

*Values were missing for 32 individuals, based on Office of Rural Health categories, 15 were categorized as RUCA 1-3; 3 as RUCA 4-6; 4 as RUCA 7-9; 1 as RUCA 10, and 9 as missing based on Rural-Urban Classifications using Rural-Urban Commuting Area Codes. The Spearman Correlation Coefficient was .49, based on 5582 observations.

© 2025 Coronado GD et al. *JAMA Network Open*.
