# Supplementary material for: Mailed Outreach and Patient Navigation for Colorectal Cancer Screening Among Rural Medicaid Enrollees: A Cluster Randomized Clinical Trial
Source: JAMA Netw Open. 2025 Mar 17;8(3):e250928. doi: 10.1001/jamanetworkopen.2025.0928 (PMC11915063; doi:10.1001/jamanetworkopen.2025.0928)
Supplement: Supplement 3. — Data Sharing Statement [file jamanetwopen-e250928-s003.pdf]

Data Sharing Statement

Coronado. Mailed Outreach and Patient Navigation for Colorectal Cancer Screening Among Rural Medicaid Enrollees. *JAMA Netw Open*. Published March 17, 2025. doi:10.1001/jamanetworkopen.2025.0928

Data
Additional Information: Registered at clinicaltrials.gov (NCT04890054) and at the NCI's Clinical Trials Reporting Program (CTRP #: NCI-2021-01032) on May 11, 2021.
Data available: Yes
Data types: Deidentified participant data, Data dictionary
How to access data: Upon manuscript acceptance, de-identified data, data dictionaries, and relevant supporting documentation from this manuscript will be made publicly available on the ACCSIS DataShare website: https://healthcaredelivery.cancer.gov/accsis/datashare/.
When available: With publication

Supporting Documents
Document types: None

Additional Information
Who can access the data: External researchers can request ACCSIS public use datasets, which are existing HIPAA-defined de-identified datasets. In general, ACCSIS public use datasets are available to anyone who requests them as long as the requestor agrees to standard data use conditions, which include agreement to comply with privacy and security notices consistent with applicable federal laws, directives, and other federal guidance.
Types of analyses: For any purpose.
Mechanisms of data availability: Via the ACCSIS DataShare website, after the requestor agrees to standard data use conditions, which include agreement to comply with privacy and security notices consistent with applicable federal laws, directives, and other federal guidance.
